# The effect of obstructive sleep apnea therapy on cardiovascular autonomic function: a systematic review and meta-analysis

**DOI:** 10.1093/sleep/zsac210

**Published:** 2022-09-15

**Authors:** Hasthi U Dissanayake, Yu Sun Bin, Kate Sutherland, Seren Ucak, Philip de Chazal, Peter A Cistulli

**Affiliations:** Sleep Research Group, Charles Perkins Centre, University of Sydney, Australia; Northern Clinical School, Faculty of Medicine and Health, University of Sydney, Australia; Sleep Research Group, Charles Perkins Centre, University of Sydney, Australia; Northern Clinical School, Faculty of Medicine and Health, University of Sydney, Australia; Sleep Research Group, Charles Perkins Centre, University of Sydney, Australia; Northern Clinical School, Faculty of Medicine and Health, University of Sydney, Australia; Centre for Sleep Health and Research, Department of Respiratory Medicine, Royal North Shore Hospital, Australia; Sleep Research Group, Charles Perkins Centre, University of Sydney, Australia; Northern Clinical School, Faculty of Medicine and Health, University of Sydney, Australia; Sleep Research Group, Charles Perkins Centre, University of Sydney, Australia; School of Biomedical Engineering, University of Sydney, Sydney, NSW, 2006, Australia; Sleep Research Group, Charles Perkins Centre, University of Sydney, Australia; Northern Clinical School, Faculty of Medicine and Health, University of Sydney, Australia; Centre for Sleep Health and Research, Department of Respiratory Medicine, Royal North Shore Hospital, Australia

**Keywords:** obstructive sleep apnea, autonomic nervous system, sympathetic activity, parasympathetic activity, cardiovascular risk, systematic review, meta-analysis, obstructive sleep apnea therapy, catecholamines, heart rate variability

## Abstract

**Study Objectives:**

Autonomic function is impaired in obstructive sleep apnea (OSA) and may mediate the association between OSA and cardiovascular risk. We investigated the effect of OSA therapy on autonomic function through a systematic review and meta-analysis of intervention studies.

**Methods:**

A systematic search using three databases (Medline, Embase, and Scopus) was performed up to December 9, 2020. Studies of OSA patients ≥ 18 years with autonomic function assessed before and after treatment with positive airway pressure, oral appliance, positional therapy, weight loss, or surgical intervention were included for review. Random effects meta-analysis was carried out for five groups of autonomic function indices. Risk of bias was assessed using the Cochrane Collaboration tool.

**Results:**

Forty-three eligible studies were reviewed with 39 included in the meta-analysis. OSA treatment led to large decreases in muscle sympathetic nerve activity (Hedges’ *g* = −1.08; 95% CI −1.50, −0.65, *n* = 8) and moderate decreases in catecholamines (−0.60; −0.94, −0.27, *n* = 3) and radio nucleotide imaging (−0.61; −0.99, −0.24, *n* = 2). OSA therapy had no significant effect on baroreflex function (Hedges’ *g* = 0.15; 95% CI −0.09, 0.39, *n* = 6) or heart rate variability (0.02; −0.32, 0.36, *n* = 14). There was a significant risk of bias due to studies being primarily non-randomized trials.

**Conclusions:**

OSA therapy selectively improves autonomic function measures. The strongest evidence for the effect of OSA therapy on autonomic function was seen in reduced sympathetic activity as assessed by microneurography, but without increased improvement in parasympathetic function. OSA therapy may reduce the risk of cardiovascular disease in OSA through reduced sympathetic activity.

## Graphical Abstract

**Graphical Abstract:**
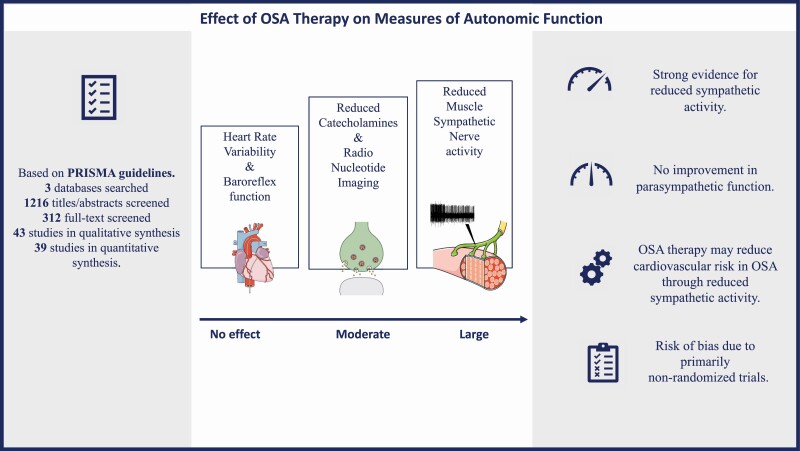

**Statement of Significance**

This study synthesizes current evidence on whether treatment for obstructive sleep apnea improves autonomic dysfunction associated with the condition. The results show that while some aspects of autonomic function are improved, others are not, and highlights the need for more rigorous studies to fully understand the impact of obstructive sleep apnea therapy on cardiovascular risk.

## Introduction

Obstructive sleep apnea (OSA) is a highly prevalent sleep disorder, estimated to affect 1 billion people globally [1]. It is characterized by repetitive periods of upper airway collapsed during sleep, resulting in intrathoracic pressure swings, repetitive oxygen desaturation, and sleep fragmentation [2]. The perturbations caused by OSA has detrimental effects on the cardiovascular system through altered cardio-metabolic pathways. Specifically, altered autonomic function plays a key role in mediating cardiovascular risk in OSA [3]. The autonomic nervous system (ANS) is a major regulator of the cardiovascular system, fundamentally through reflex arcs that modulate heart rate and blood pressure. The parasympathetic nervous system decreases heart rate and cardiac contractility, whilst sympathetic modulation opposes these effects and increases peripheral vasoconstriction [3].

Patients with OSA display a cyclical pattern of heart rate and blood pressure surges associated with activation of the sympathetic and parasympathetic nervous system from repetitive apnea events. Altered baroreceptor and chemoreceptor reflexes associated with elevated sympathetic nerve activity may contribute to increased cardiovascular risk in OSA. Several methods are utilized to measure autonomic function in OSA, each with advantages and limitations. Our recent systematic review, found evidence of altered autonomic function in OSA, specifically elevated sympathetic nerve activity [4]. Elevated sympathetic nerve activity is a hallmark of hypertension, a major risk factor for cardiovascular disease. Altered autonomic function may be a key mediator linking OSA and cardiovascular disease and mitigating these autonomic changes by treatment of OSA may reduce cardiovascular risk.

Positive airway pressure (PAP), oral appliance (OA), and positional therapy are the recommended treatment options for OSA. Whereas surgical interventions such as bariatric surgery, oro-pharyngeal surgery, and hypoglossal nerve stimulation are studied in highly selected OSA patients, lifestyle interventions such dietary control and exercise are recommended to mostly overweight or obese patients with OSA. PAP and OA have been shown to ameliorate OSA and improve health-related quality of life and blood pressure, however the effect of OSA therapy on autonomic function remains unclear [5]. Accordingly, the aim of this systematic review is to synthesize evidence from available studies on the effectiveness of obstructive sleep apnea therapy on autonomic function.

## Methods

### Protocol and registration

The design of this systematic review and meta-analysis is registered in PROSPERO (CRD42019146171), the international prospective register of systematic reviews.

### Eligibility criteria

Original research studies were considered eligible for inclusion in this review if they met the following criteria:

Population: adults ≥ 18 years of age with a diagnosis of OSA, defined as an apnea hypopnea index (AHI) ≥ 5/h or respiratory disturbance index (RDI) ≥ 5/h.

Intervention: Any intervention for OSA, including CPAP, OA, positional therapy, weight loss, and surgical interventions to improve upper airway patency.

Comparison: Presence of a control or comparison group without OSA treatment. This could include participants at baseline before the intervention (within group comparison), or a comparison group of participants who did not receive intervention (between group).

Outcome/s: Autonomic function outcomes included were measures of Heart Rate Variability (HRV), Blood Pressure Variability (BPV), Baroreceptor function, Catecholamines, Muscle Sympathetic Nerve Activity (MSNA), and Skin Sympathetic Response (SSR). Autonomic function measurements could be made in the wake resting subject, during sleep, or resting wakefulness before or after sleep. Furthermore, we included autonomic function measurements across varying lengths of analysis, specifically for HRV. Definitions and indices of different aspects of autonomic function and typical associations with health outcomes are described in Appendix 1. Study design: Randomized control trials (RCTs) and before and after studies among patients who received OSA therapy were eligible for inclusion. The primary outcome of interest was change in autonomic function associated with intervention.

Language: English language.

### Data sources and search strategy

A literature search using the databases of Medline via OvidSP, Embase classic via OvidSP, and Scopus was performed up to December 9, 2020 and all prior years of publication were considered. The search strategy involved the following keywords: sleep apnea, obstructive sleep apnea, hypopnea, disordered breathing, OSA, and OSAS syndrome, autonomic nervous system, sympathetic, parasympathetic, electrocardiography, heart rate, baroreflex, pressoreceptors, and blood pressure. Sleep apnea related keywords included alternate spellings and were combined with autonomic function related keywords. Search results were filtered for studies in adult humans only. The exact search strategy for each database can be found in Appendix 2. All search results were exported into Endnote literature management software, Endnote X9 (Clarivate Analytics, Philadelphia). Duplicate records were excluded using the automated function in Endnote based on matching authors, article title, and journal title. Review articles were excluded through filtering by “Article Type” within Endnote.

### Study selection

**Figure 1.**
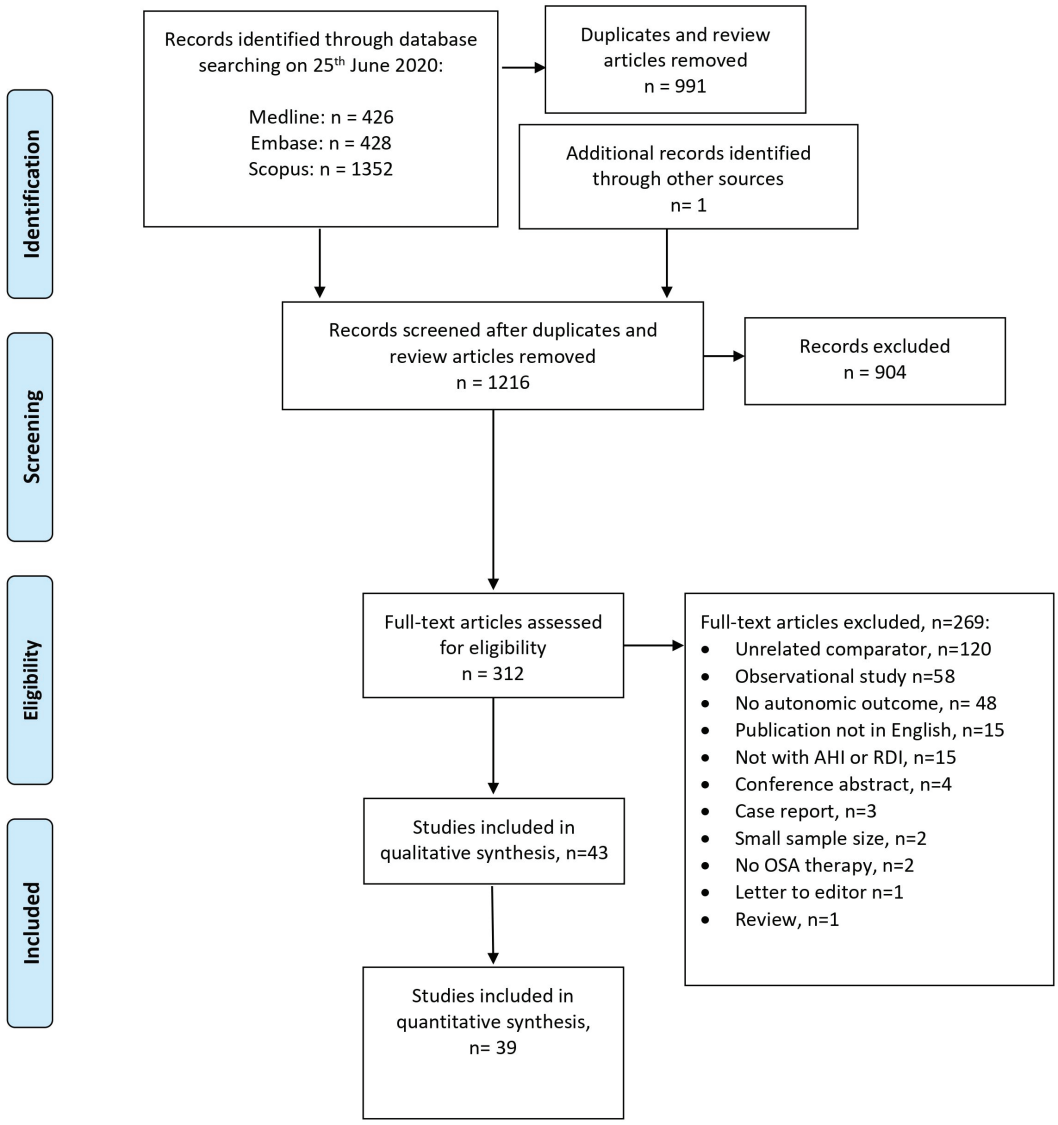
Study selection flowchart.

Study titles and abstracts were screened by two independent reviewers (KS and SU) and any discrepancies resolved by a third reviewer (HD). Full-text review was conducted by a third reviewer (HD). Study selection is described in Figure 1.

### Data extraction

Full-text review and data extraction from each article was completed by one reviewer (HD) and independently checked by another (YSB). Data extracted for meta-analysis included index of autonomic function, unit of measurement, time of measurement (e.g. during sleep stage or during wake), mean and standard deviation (SD) with and without OSA treatment, the number of patients in treated and untreated groups, treatment characteristics (type, dose, duration, and adherence), and characteristics of study participants (baseline OSA severity as indicated by AHI or RDI, proportion of male participants, and mean age of participants). Medians and interquartile ranges, standard errors, and 95% confidence intervals were extracted if means and SDs were not reported. For cross-over studies involving two active interventions, data from both interventions were included for analysis. Where studies reported results for two groups of participants e.g. those adherent to treatment and those not adherent, both groups were included for analysis.

### Risk of bias assessment

The Cochrane Collaboration tool for assessing risk of bias was utilized. This is a two-part tool, addressing the seven specific domains for risk of bias, namely, sequence generation, allocation concealment, blinding of participants and personnel, blinding of outcome assessment, incomplete outcome data, and selective outcome reporting. The first part of the tool describes what was reported to have happened in the study, in sufficient detail to support a judgement about the risk of bias. The second part of the tool assigns a judgement relating to the risk of bias for that entry. This is achieved by assigning a judgement of “Low risk” of bias, “High risk” of bias, or “Unclear risk” of bias. Risk of bias assessment was carried out by two independent reviewers (KS, YSB) with any disagreement independently resolved by a third reviewer (HD). The results of the risk of bias assessment are included in Appendix 3.

### Statistical analysis

Meta-analysis was carried out using Stata SE 16.1 for Windows (StataCorp, College Station, TX, United States). Effect sizes (Hedges’ *g*) was calculated using the data from included studies. Given that most studies assessed autonomic function using more than one metric, we grouped the outcomes and conducted meta-analysis for each group of outcomes separately: (1) Heart rate variability (HRV), (2) Baroreceptor function, (3) Catecholamines, (4) Muscle sympathetic nerve activity (MSNA), and (5) I-Metaiodobenzylguanidine (MIBG). No meta-analysis was conducted for measures of mechanical effects of respiration since there was only one study reporting this outcome [6].

The heterogeneity of effect sizes was evaluated using Cochran’s *Q* statistic (*p* < .05) and the *I*^2^ statistic, with values of 25%, 50%, and 75% representing low, medium, and high heterogeneity respectively [7]. Effect sizes were pooled using a random effects model (DerSimonian-Laird). Forest plots were produced to visually summarize the results. A funnel plot was used in conjunction with Eggers’ test to examine the possibility of publication bias [8] (Appendix 4). We did not rely on Eggers’ test which has lower power to detect biases, especially when there are fewer than 10 studies. The trim-and-fill method was used to balance funnel plot asymmetry [9].

We planned to examine the impact of mode of therapy, duration of therapy, adherence to therapy, and baseline OSA severity on the outcomes through a meta-regression, however, we found this was not possible after data extraction due to a lack of suitable data. First, the autonomic function measures are heterogeneous and cannot be justifiably combined into one overall outcome. Second, many studies reported multiple autonomic function outcomes and are already included in more than one of the outcome-specific analyses. Third, many studies did not report adherence to therapy (*n* = 15) and together with missing data on duration of therapy, baseline OSA, and patient demographics, less than half of the relevant studies for each outcome had complete data for multivariate meta-regression (Appendix 5). We report the results for univariate and multivariate meta-regression analyses in Appendix 5 but note that these results could be misleading.

## Results

### Study characteristics

A total of 43 studies were included in this review (Table 1). Of these, 31 studies compared autonomic function in 649 patients before and after PAP therapy, two studies were conducted in 22 patients before and after MAD [10, 11] and one study in 40 patients before and after uvulo-palato-pharyngoplasty or Z-palatopharyngoplasty surgery [12].

**Table 1.** Characteristics of included studies which evaluate the impact of OSA intervention on autonomic function (*N* = 43)

Study	Study design, intervention type, and duration, and compliance	Participants *N*, % male, and age (mean, *SD*)	Baseline and treated AHI/AI/RDI (mean events/h, *SD*)	Inclusion and exclusion criteria	Autonomic measure/s (categories)
Roche *et al*. [38]	Before and during 1 night of CPAP (within subjects).	*N* = 38 (89.5% male), 52.2 years (11.2).	Baseline AHI mean 56.9 (*SD* 28.4). Treated AHI mean 3.8 (*SD* 5.4).	*Excluded:* Permanent or paroxysmal atrial fibrillation, complete bundle branch blocks, shy-drager syndrome, use of antiarrhymic agents, and permanent venticular or atrial pacing.	HRV
Wu *et al*.[39]	Before and during 1 night of CPAP (within subjects).	*N* = 13, gender not stated, 53.8 years (*SD* 4.0).	Baseline AHI mean 62.7 (*SD* 5.8) Treated AHI not stated.	*Excluded*: history of cardiac arrhythmias, congestive heart failure, myocardial infarction, ischemic heart disease, stroke, diabetes mellitus, central sleep apnea, restless legs syndrome, periodic limb movement disorder, chronic obstructive pulmonary disease.	HRV
Kufoy *et al*. [24]	Before and during 1 night of PAP (within subjects).	*N* = 39 (62% male), 51.4 years (*SD* 7.4).	Baseline AHI mean 47.7 (*SD* 15.1) Treated AHI not stated.	*Excluded:* history of cardiovascular or cerebrovascular disease, sleep disorders, thyroid or endocrine disease including diabetes, use of antiarrhythmic, anticholinergic, or antidepressant medications.	HRV
Sukegawa *et al*. [40]	Before and during 1 night of CPAP (within subjects).	*N* = 17 (100% male), 53.7 years (*SD* 13.5).	Baseline AHI mean 42.5 (*SD* 25.9). Treated AHI mean 9.3 (*SD* 7.9).	Not stated.	Catecholamines
Palma *et al*. [41]	Before and during 1 night of CPAP (within subjects). Before and after 2 years of CPAP (within subjects): Moderate OSA: CPAP use 2.1 (*SD* 0.8) years Severe OSA: CPAP use 1.9 (*SD* 0.7) years. Only compliant i.e. ≥4 h/night on 70% of nights included in study.	Moderate OSA: *n* = 16 (81% male), 51.8 years (*SD* 13.4). Severe OSA: *n* = 14 (79% male), 52.3 years (*SD* 11.9).	Moderate OSA: Baseline AHI mean 26.6 (*SD* 1.8). Acute treated AHI mean 4.2 (*SD* 6.4). Chronic treated AHI mean 7.6 (*SD* 3.9). Severe OSA: Baseline AHI mean 55.8 (*SD* 16.5). Acute treated AHI mean 3.9 (*SD* 8.1). Chronic treated AHI mean 6.1 (*SD* 3.4).	*Excluded:*Psychiatric, craniofacial, respiratory or neurologic disorders; history of diabetes mellitus, coronary artery disease, arrhythmias, heart failure, or ST-T wave abnormalities; presence of other sleep disorders; smokers; patients taking cardiovascular medications, or medications known to affect the ANS.	HRV
Jennum *et al*. [42]	Before and after 8 days of PAP (within-subjects). Compliance not stated.	*N* = 14 93% male 42 years (range 36–66).	Baseline AHI mean 54.6 (*SD* 1.7). Treated AHI mean 13.2 (*SD* 15.8).	*Included:* apnea index > 30/h, mean apnea time index > 20 s. *Excluded:* On medications during last half year, known medical or psychiatric disorders except hypertension.	Catecholamines
Otsuka *et al*. [29]	Before and after 1 month of nCPAP treatment (within subjects). Compliance self-reported as “good”.	*N* = 8 Gender not stated. Age not stated.	Baseline AHI mean 86.3 (*SD* 29.3). Treated AHI mean 5.4 (*SD* 3.5).	All patients were obese. *Excluded:*chronic obstructive pulmonary disease, primary cardiac diseases, daytime hypertension, antihypertensive medications, diabetes, hyper-and hypothyroidism, diseases of the liver and central nervous system.	Iodine-123-MIBG imaging
Heitmann *et al*. [43]	Before and after 41.6 ± 16.9 days of CPAP (within subjects). Compliance 5.7 (*SEM* 0.7) h/night.	*N* = 18 (gender not stated). Normotensive: 8 (gender not stated), 52.6 years (*SD* 10.1). Hypertensive: 10 (gender not stated), 47.9 years (*SD* 10.6).	Normotensive: Baseline AHI mean 42.8 (*SD* 20.0). Treated AHI 0.3 (*SD* 0.7). Hypertensive: Baseline AHI mean 70.3 (*SD* 28.9). Treated AHI 0.4 (1.0).	*Excluded*: systolic blood pressure > 220 mmHg or diastolic blood pressure > 110 mmHg; myocardial infarction; stroke; heart failure; neurological disease; or a history of alcohol or drug abuse; antihypertensive medication was discontinued at least 3 weeks before the study began.	Catecholamines
Schytz *et al*. [22]	Before and after 2 months or more of CPAP (within subjects). Days of CPAP ≥ 4 h 82 days (*SEM* 4 days). Days of usage 67 days (*SEM* 1 day).	OSA patients *n* = 14, gender not stated, age not stated.	Baseline AHI and treated AHI not stated.	*Excluded:* history of stroke, myocardial infarction, stenosis of carotid arteries (>50%), unstable chronic lung disease, anemia, present cardiac arrhymia, central sleep apnea, REM sleep behavior disorder, narcolepsy, medication with effects on central nervous system.	Sympathetic activity measuredwith near-infrared spectroscopy (NIRS).
Tasali *et al*. [44]	Before and after 8 weeks of CPAP treatment (within subjects). CPAP compliance 6.6 h (*SEM* 0.4 h). Days not used over 8 week treatment period 3.4 days (*SEM* 1.2)Yy031272.	*N* = 9 (0% male), 30.6 years (*SD* 1.7).	Baseline AHI mean 24.3 (*SEM* 5.5). Treated AHI mean 2.0 (*SEM* 1.0).	All participants have polycystic ovary syndrome.	HRV
Quadri *et al*. [45]	Before and after 2 months of CPAP (within subjects). Compliance not stated.	*N* = 12 (67% male), 58.0 years (*SD* 9.7).	Baseline AHI mean 45.4 (*SD* 14.9). Treated AHImean 7.1 (*SD* 7.7).	*Excluded:*Presence of cardio-vascular, neurological, metabolic, and orthopedic co-morbidities and the use of beta-blockers therapy or any drug able to modify autonomic system function.OSAH treatment (bite, continuous positive airways pressure, surgery).	HRV
Roche *et al*. [46]	Before and after 3 months of CPAP (within subjects) Compliance not stated.	*N* = 14 (86% male) 61.4 years (*SD* 8.1).	Baseline AHI mean 50.6 (*SD* 13.7). Treated AHI mean 2.2 (*SD* 2.5).	*Excluded:* coronary artery disease, electrical left ventricular hypertrophy or ST-T wave abnormality, congestive heart failure, diabetes mellitus, beta blockers, antiarrythmic drugs, and digitalis.	HRV
Glos *et al*. [16]	Randomized cross-over trial of MAS and CPAP. Before and after 12 weeks of CPAP (within-subjects). Before and after 12 weeks of MAS (within-subjects). Compliance not stated.	*N* = 40 (83% male), 49.5 years (*SD* 11.8).	Baseline AHI mean 28.5 (*SD* 16.5). MAS treated AHI mean 13.7 (*SD* 12.0). CPAP treated AHI mean 3.5 (SD 5.2).	*Excluded:* drug abuse, medication that influences sleep, presence of other sleep disorders, history of any OSA treatment, psychiatric or neurological diseases, atrial fibrillation, any medication that could affect heart rate, craniomandibular disorders, acute/subacute dental conditions that affects treatment with mandibular advancement device, discontinuation of therapy for more than 1 week.	HRV Baroreflex function
Belozeroff *et al*. [6]	Before and after 6 months of CPAP (within subjects). Compares those compliant (>3 h/night; mean 6.2 h, SEM 0.9) and those non-compliant (<3 h/night, mean 0.7 h, *SEM* 0.2).	*N* = 13 (100% male) Compliant: *n* = 6, (100% male) 48.5years (*SE* 4.2). Non-compliant: *n* = 7, (100% male) 44.1 years (*SE* 3.7).	Compliant: Baseline AHI mean 87.8 (*SE* 15.6). Treated AHI not stated. Non-compliant: Baseline AHI mean 53.8 (*SE* 11.6) Treated AHI not stated.	*Excluded:* diabetes, cardiac arrhythmia, congestive heart failure, lung disease.	HRV Baroreflex function
Fatouleh *et al*.[47]	Before and after 6 months, and after 12 months of CPAP (within subjects). Compliance not stated.	*N* = 21 at baseline (86% male), 55 years (*SD* 2). *N* = 16 at 6 months (81% male), age not stated. *N* = 15 at 12 months (97% male), age not stated.	Baseline AHI mean 44 (*SD* 5). Treated AHI not stated.	Not stated.	MSNA
Fatouleh *et al*. [48]	Before and after 6 months of CPAP (within subjects). Compliance 6.0 (*SEM* 0.4) h/night.	*N* = 11 (85% male), 53 years (*SEM* 3).	Baseline AHI mean 38 (*SEM* 5). Treated AHI mean 4 (*SEM* 2).	Not stated.	MSNA
Lundblad *et al*. [49]	Before and after 6 months of CPAP (within subjects). Compliance 5.0 (*SEM* 0.4) h/night	*N* = 13 (77% male), 54 years (*SE* 3).	Baseline AHI mean 41 (*SEM* 4). Treated AHI mean 3 (*SEM* 2).	Not stated.	MSNA
Ferland *et al*. [18]	Pragmatic trial of sibutramine vs. CPAP. Before and after 1 year of sibutramine (within subjects). Compliance 91.7% (not defined). Before and after 1 year of CPAP(within subjects). Compliance 100% (not defined).	*N* = 40 (88%). Sibutramine: *n* = 19 (86% male), 49 years (*SD* 9). CPAP: *n* = 16 (88% male), 49 years (*SD* 9).	Sibutramine: Baseline AHI mean 54 (*SD* not stated). Treatment-baseline difference in AHI: −3 (SD 6). CPAP: Baseline AHI mean 52 (*SD* not stated). Treatment-baseline difference in AHI: −40 (*SD* 30).	*Included:* untreated OSA, controlled systemic hypertension, type 2 diabetes, dyslipidaemia and/or visceral obesity. *Excluded:* uncontrolled systemic hypertension (>145/90mmHg), previous pharmacological or surgical treatment for weight loss, previous CPAP, severe diurnal hypersomnolence requiring immediate treatment.	HRV
Shiina *et al*. [50]	Before and after 3 months of CPAP therapy.	*N* = 50 (90% Male), 54 years (*SD* 10).	Baseline AHI 54 (*SD* 22). After CPAP AHI not stated.	Inclusion: patients with moderate to severe OSA. Exclusion: coronary artery disease, receiving insulin, receiving beta blockers.	HRV Baroreceptor function
Waravdekar *et al*. [51]	Before and after 1 month compliance CPAP therapy (4.7 + −2.4 h/night).	*N* = 7 (86% male), 50 years (*SD* 11).	Baseline AHI mean 69 (*SD* 40). CPAP AHI not stated.	*Included:* untreated OSA, all subjects were overweight (BMI 38.0 ± 5.1), snored, excessive daytime sleepiness, medications wer enot withheld or changed during the study period. *Subject characteristics:* 1× noninsulin-dependent DM and treated with oral hypoglycemic agent. 2× hypertensive treated with an angiotensin-converting enzyme inhibitor.	MSNA
Bakker *et al*. [20]	Comparison of weight loss surgery and CPAP after 6, 12, 18 months of intervention (between subjects). CPAP usage not stated.	Weight loss surgery: *n* = 12 (17% male), 43 (IQR 37, 49) years. CPAP: *n* = 15 (73% male), 48 (IQR 37, 52) years.	Weight loss surgery: AHI at baseline 18.1 [IQR 16.3, 67.5], 6 months 10.5[5.0, 20.8], 12–18 months 6.5 [1.9, 12.8]. CPAP: Baseline AHI 36.5 [IQR 24.7, 77.3], 6 months 5.3 [1.6, 10.9], 12–18 months 3.8 [1.2, 10.6].	*Included:*BMI ≥ 30, free from cardiovascular co-morbidities. *Excluded:* smoking,presence of any cardiopulmonary, endocrine, or sleep disorders (other than OSA, consumption of any medications that could affect either cardiopulmonary function or sleep, including antihypertensive.	HRV
Berger *et al*. [21]	Comparison of exercise training and no exercise training after 9 months of intervention (between subjects).	Exercise training: *n* = 36 (67% male), 52 [IQR 60–64] years. Control: *n* = 38 (58% male), 62 [IQR 60–65] years.	Exercise training: AHI at baseline 22 (*SD* 7.0), 9 months 17 (*SD* 10). Control: AHI at baseline 21 (*SD* 6), 9 months 22 (10).	*Included:* Cardiovascular history, hypertension, diabetes mellitus, current smoker, alcohol use, current medication (beta-blockers, other antihypertensive agent, antithrombotic agent, lipid-lowering agent, insulin or antidiabetic oral medication, psychotropic agents, hypothyroidism agent). *Excluded:* current OSA treatment, cardiovascular or respiratory comorbidities, and/or excessive daytime sleepiness justifying immediate initiation of CPAP, respiratory or heart disease contraindicating exercise discovery during stress testing, Parkinson’s disease.Frequent ectopic beats (>0.7%).	HRV
Bonsignore *et al*. [52]	Comparison of before and after 3–14months of CPAP therapy. Average CPAP nightly 5 (*SD* 2) h. Mean treatment duration 6 (*SD* 4) months.	*N* = 10 (100% Male), 47 (*SD* 9) years.	Baseline AHI 82 (*SD* 14). CPAP withdrawal AHI 63 (*SD* 26).	*Inclusion:*office blood pressure > 140/90 mmHg, no clinical or laboratory evidence of chronic heart failure, hypertension, or other diseases causing autonomic dysfunction, no treatment with cardiovascular drugs, smokers. *Exclusion:* reported habitual alcohol intake of more than 30 g/day or drug use.	Baroreflex function
Chang *et al*. [53]	Before and after 3 months of CPAP therapy. CPAP compliance was defined as days of CPAP use (for at least 4 h/d) and was expressed as a percentage of the total study period = 78.9 (*SD* 14.5).	*N* = 13 (100% male), 50 (*SD* 7) years.	Baseline AHI: 60.3 (*SD* 21.2). CPAP treated AHI: 4 (*SD* 2).	*Inclusion:*middle-aged untreated men with severe OSA. *Exclusion:* clinical diagnosis or history of respiratory disease, cerebrovascular or coronary heart disease, endocrinologic conditions (e.g. neurodegenerative diseases, epilepsy, head injury), psychiatric disorders (e.g. recurrent depression, psychotic disorders, substance-related disorders), or current intake of psychotropic medications.	HRV
Chrysostomakis *et al*. [54]	Before and after 2 months of CPAP therapy. CPAP compliance not stated.	*N* = 26 (69% male), 49 (*SD* 8) years.	Baseline AHI: 58 (*SD* 24). CPAP treated AHI: not stated.	*Inclusion:*documented moderate to severe sleep apnea. *Exclusion:* hypertension, diabetes mellitus, sinus node disease or atrioventricular conduction abnormalities, indications of coronary artery disease, dilated or hypertrophic cardiomyopathy, valvular heart disease, respiratory failure and lung disease that might have led to structural or functional pulmonary dysfunction, patients currently on medication with cardioactive drugs (affecting the heart rate), hypnotics or drugs affecting sleep.	HRV
Coruzzi *et al*. [10]	Before and after 3 months of oral device treatment. Oral device treatment compliance not stated.	*N* = 10 (60% male), 48 (*SD* 10) years	Baseline AHI: 18 (*SD* 1). CPAP treated AHI: 4 (*SD* 1).	*Inclusion:* normotensive, free of any other known disease, receiving no medications, nonsmokers at the time of the study and during the previous 6 months. All subjects were used to drinking no more than 2 cups of expresso coffee per day, this being the case both at baseline and during treatment. *Exclusion:*participants with no evidence of significant occlusion defects.	HRV
Huang *et al*. [12]	Before and after uvulo-palato-pharyngoplasty or Z-palatopharyngoplasty.	*N* = 40 (% sex not stated), age not stated.	Before surgery AHI: 35 [IQR 17, 59]. After surgery AHI: 15 [4, 43].	*Inclusion:* patients with OSA, surgical procedure were determined upon the discretion of the treating sleep surgeon based on the severity of OSA with PSA and conditions of upper airway abnormality as examined by flexible fibroscopy. *Exclusion:* patients with moderate-to-sever heart failure, any type of arrhythmia, pacemaker implantation, underlying medical diseases known to affect autonomic system, such as diabetes mellitus and chronic renal failure; had neoplastic disorders; had stroke history, critical carotid stenosis requiring carotid end-arterectomy or stenting, myocardial infarction, or coronary artery diseases status post percutaneous trans-luminal coronary angioplasty or bypass surgery; and had central or peripheral disorders known to affect autonomic system, such as Parkinson’s disease, diffuse Lewy-body disease, multiple system atrophy, and pure autonomic failure.	HRV Baroreflex function
Isobe *et al*. [55]	Before and after average 123 days of nCPAP therapy. nCPAP mounting time 5 (SD 2) h/day.	*N* = 76 (83% Male), 62 (IQR 25–88) years.	Baseline AHI: 45 [7–141]. CPAP treated: 3 [0.3–23].	*Inclusion:*indicated for subjects with moderate to severe OSAS, defined as an AHI ≥ 20 (events/hour) with strong subjective symptoms, such as excessive daytime sleepiness, and morningheadache. *Exclusion:* patients with > 5 central sleep apnea events per hour who could not undergo nCPAP.	MSNA
Ito *et al*. [19]	Before and after nCPAP Before and after uvulopalatopharyngoplasty.	*N* = 5 (nCPAP), *n* = 2 (uvulopalatopharyngoplasty), (100% male), 46 [range 32–59] years.	Baseline AHI: 42 [range 31–77] Treated AHI: 2 [range 1–4].	*Inclusion:* newly diagnosed OSA, never been treated for OSA, median BMI 32.2 kg/m^2^ start of the study. History of hypertension (*n* = 5), none presented clinical or electrocardiographical evidence of coronary artery disease, left ventricular hypertrophy, or ST-T wave abnormality; none suffered from congestive heart failure or diabetes mellitus, and none received any medication including b-blocker, an antiarrhythmic drugs, or digitalis.	Baroreflex function HRV
Jurysta *et al*. [56]	Before and after 4 years of nCPAP therapy. Compliance 6 ± 1 h/night.	*N* = 8 (100% Male), 46 (*SD* 7) years.	Baseline AHI: 64 (*SD* 23) nCPAP treated AHI: 6 (*SD* 4).	*Inclusion:* patients suffering from severe SAHS and treated by nCPAP during at least one year were selected retrospectively, provided they did not develop any cardiovascular disease or mental or neurological disorder. Exclusion: other sleep disorders such as parasomnia.	HRV
Kuramoto *et al*. [57]	Before and after 3 months nCPAP therapy. Treatment compliance not stated.	*N* = 38 (87% male), 52 (*SD* 13) years	Baseline AHI: 57 (*SD* 21). nCPAP treated AHI: not stated	*Inclusion:* patients diagnosed with OSA by polysomnography. Exclusion: Individuals with cerebral infarction, myocardial infarction within 3 months, coronary bypass graft, aortic diseases and atrial fibrillation.	HRV
Limphanudom *et al*. [58]	Before and after 1, 3, and 6 months CPAP therapy. Compliance 5 h/night.	*N* = 10, (100% male), 45 (*SD* 5) years.	Baseline AHI: 61 (*SD* 32). CPAP treated AHI not stated.	*Inclusion:* newly diagnosed moderate to severe OSA. None had any underlying disease that was causing autonomic dysfunction and cardiac arrhythmia, no evidence of chronic lung disease, or any beta-blocker or anti-arrhymic drugs.	HRV
Nakamura *et al*. [59]	Before and after 1 month of nCPAP therapy.	*N* = 48 (94% Male), 46 (*SD* 11) years.	Before treatment AHI: 52 (*SD* 19) After 1 month after of CPAP treatment AHI: not stated	*Inclusion:* consecutive OSAHS with an AHI ≥ 20 who had undergone nCPAP therapy. None of the participants had cardiac disease or arrhythmia, 16 patients had hypertension and were on various antihypertensive agents for > 1 month before the start of this study and throughout the study. None of the patients had received antiarrhythmic agents. *Exclusion:*not stated.	I-metaiodobenzyl-guanidine (MIBG) imaging
Narkiewicz *et al*. [60]	Before and after 1month, 6 months and 1 year of CPAP therapy. Compliance with prescribed CPAP treatment was based on self-reported estimated use, expressed as average time with CPAP in relation to total time spent in bed. All treated patients reproted > 75% compliance with CPAP.	*N* = 11 (91% Male),46 (*SD* 8) years.	Baseline AHI:27 (*SD* 6).	*Inclusion:* normotensive, free of any other known diseases, receiving no medication, no central sleep apnea. *Exclusion:*not stated.	MSNA
Nelesen *et al*. [13]	Randomized control trial comparing 1 night and 1 week of CPAP treatment with placebo (ineffective level of CPAP). CPAP usage 6 (*SEM* 0.2) h/night. Placebo usage 6 (*SEM* 0.2) h/night.	CPAP: N = 23 (74% Male), 47 (*SEM* 2) years. Placebo: *N* = 18 (89% Male), 50 (*SEM* 2) years.	CPAP RDI 56 (*SEM* 5). Placebo RDI 39 (*SEM* 5).		
Noda *et al*. [15]	Randomized control trial comparing 3 months of CPAP treatment with no treatment (between subjects).	CPAP *N* = 14 (100% Male). Non-CPAP *N* = 19 (100% Male). *Average age of both groups 53 (*SD* 10) years. Average in each group not provided.	CPAP: Baseline AHI 53 (*SD* 12). During CPAP AHI 9 (*SD* 4). Non-CPAP AHI data not provided. *No differences in baseline AHI were seen between groups. Average AHI values not provided.	*Inclusion:*Male patients with OSAS, aged 28 to 71 years. *Exclusion:*diabetes mellitus, chronic obstructive lung disease, coronary or valvular heart disease, congestive heart failure, renal failure, or endocrine dysfunction, antihypertensive agents at the time of enrollment.	Baroreflex function Catecholamines
Shiomi *et al*. [11]	Before and after mean 3.3 ± 1.3 months of mandibular advancement therapy. Treatment compliance not stated.	*N* = 12 (83% Male), 53 (*SD* 11) years.	Baseline RDI 30 (*SD* 17). After mandibular advancement RDI 9 (*SD* 10).	*Inclusion:* normal spirometry and arterial blood gases, normal Valsalva ratios, sufficiently healthy teeth to anchor the dental appliances. *Exclusion:*evidence of autonomicdysfunction or neuropathy, clinical presence of neurological disease, or lung disease.	HRV
Somers *et al*. [61]	Before and during 2.1 ± 0.5 h of CPAP treatment.	N = 4 (100% Male), 44 (*SD* 12) years.	Baseline AHI 65 (*SD* 9) during CPAP therapy AHI no stated.	*Inclusion:*hypertension. Other inclusion exclusion criteria not stated.	MSNA
Tamisier *et al*. [62]	Before and after 6 months of CPAP therapy. Average compliance 4.5 h/night.	*N* = 26 (% gender not stated), 24–76 (range) years.	Baseline AHI 34 (*SD* 20). Post CPAP AHI 5 (*SD* 5).	Inclusion: 24–76 year olds reporting to a sleep clinic with the complaint of snoring and or sleepiness and confirmation of sleep apnea on polysomnography. No other comorbidities.	MSNA
Yamaguchi *et al*. [63]	Before and after CPAP use. *Compliance not stated.	*N* = 28 (93% Male), 62 (*SD* 10) years.	Baseline AHI 45 (*SD* 23). Post CPAP AHI 2 (*SD* 2).	*Inclusion:* no comorbidities, malignancy in any organ, sever hypertension, severe diabetes mellitus, conspicuous heat failure, heart or cerebral attack, renal failure, or impaired cognatic function. *Exclusion:* taking agonists, b antagonists, or anticholinergic agents, as well as those with atrial fibrillation or artificial cardiac rhythm generated by a pacemaker, were excluded from the analysis.	HRV
Ziegler *et al*. [14]	Randomizedplacebo control trial comparing before, 2 days and 10 days of CPAP or Placebo (CPAP at ineffective pressure) treatment. *Compliance not stated.	CPAP: *N* = 20 (70%Male), 48 (*SD* 1) years. Placebo CPAP: *N* = 18 (89% Male), 50 (*SD* 2).	CPAP RDI 54 (*SD* 5). Placebo RDI 39 (*SD* 5).	*Inclusion:* no previous CPAP treatment, between 35 and 65 years, from 100 to 170% of ideal body weight. *Exclusion:* major medical disorder other than hypertension and sleep apnea.	Catecholamines
Marrone *et al*. [23]	Before and during CPAP therapy.	*N* = 10 (90% Male), 49 (*SE* 4) years.	Without CPAP AHI 75 (*SE* 5). With CPAP AHI 10 (*SE* 2).	*Inclusion:* affected by OSAS, *n* = 1 mildly hypertensive participant, none receiving antihypertensive treatment. *Exclusion:* not stated.	Catecholamines
Dal-Fabbro *et al*. [17]	Randomized cross over trial comparing before and after 1 month of placebo oral appliance (POA), mandibular advancement device (MAD), and CPAP therapy.	*N* = 23 (83% Male), 47 (*SD* 9) years.	Baseline AHI 42 (*SD* 5). With POA AHI 49 (*SD* 6). With MAD AHI 27 (*SD* 5). With CPAP AHI 3 (*SD* 0.4).	*Inclusion:* moderate to severe OSA (AHI ≥ 20), either gender, BMI under 35 kg/m^2^, 25–65 years of age, dentition in good condition, and a minimum mandibular protrusion of 7 mm. *Exclusion:* periodontal disease, severe temporomandibular disorders, other sleep disorders, disturbances that interfere in CPAP use, alcohol or drug abuse, previous OSA treatment.	HRV

Numbers refer to the number of participants with OSA (not the whole sample being studied in each study). AHI = apnea-hypopnea index. AI = apnea index. CPAP = continuous positive airway pressure. PSG = polysomnography. SD = standard deviation. SEM = standard error of the mean.

Three randomized control trials were included: two studies that compared CPAP vs placebo in 79 patients [13, 14] and one study that compared CPAP vs no CPAP in 33 patients [15]. Furthermore, two randomized cross-over trials were included: one study of MAD and CPAP 40 patients [16] and one study of MAD and placebo MAD in 23 patients [17].

Additionally, there was one nonrandomized trial of sibutramine vs CPAP in 40 patients [18], three prospective cohort studies were included, one study of CPAP and uvulopalatopharyngoplasty surgery in seven patients [19], one study of weight loss surgery and CPAP in 27 patients [20], one study of physical activity program vs no physical activity program in 74 patients [21]

### Risk of bias assessment of included studies

Risk of bias assessment is shown in Supplementary Materials (Supplementary Figure S1a and b). We found a high risk of bias in allocation concealment and random sequence generation because most of the studies reviewed were not randomized trials. Furthermore, studies that were randomized trials, the method used for randomization or how the randomization sequence was concealed was not described. We found low risk of bias for the blinding of participants and personnel and for the blinding of outcome assessment. This was predominantly driven by the nature of our outcome measures, that is, objective measures that are scored by automated algorithms and which are unlikely influenced by performance or detection bias. Furthermore, we found low risk of bias for incomplete outcome data and selective reporting indicating little evidence for attrition bias and reporting bias.

### Meta-analysis

Forty-three studies were included in this review however, only *n* = 38 studies were considered for meta-analysis due to four studies including only graphical results [13, 15, 22, 23] and one study not reporting standard deviations for the outcome data [24].

### Heart rate variability

Heart rate variability (HRV) is a noninvasive measure of cardiac autonomic function which can be evaluated by a number of analysis methods including; time domain, frequency domain, and non-linear methods [25]. The analysis of the beat-to-beat variation in heart beats provide an indirect measure of sympathetic and parasympathetic modulation of the sinus node [25]. There were 24 studies investigating the impact of OSA treatment on HRV using one or more of these methods. Since studies typically included multiple measures of HRV evaluated by a number of methods. To avoid double-counting of results within a study, we grouped HRV outcomes into the five categories: (i) global (*n* = 14 studies), (ii) sympathetic (*n* = 14), (iii) parasympathetic (*n* = 19), (iv) low frequency (*n* = 11), and (v) very low-frequency measures (*n* = 6); and selected the most common or the most directly comparable measure in each group for comparison across studies. These categories are further defined in Appendix 1.

### (i) Global HRV

**Figure 2.**
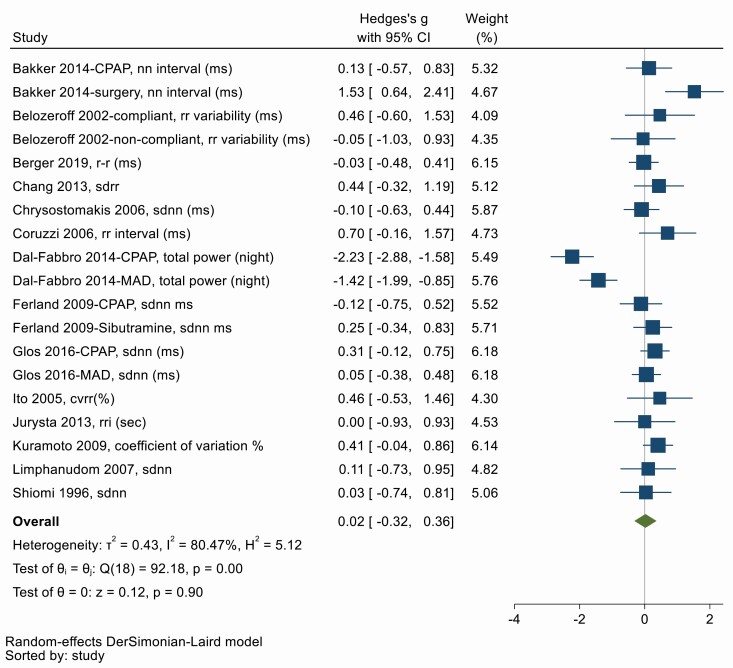
Forest plot for effect of OSA treatment on global measures of heart rate variability (*n* = 14 studies).

CoV (%), CVRR, mean RR, mean NN, NN interval, RRI, RR interval variation, RR variability, SD, SDNN, SDaNN, SDIndex, SDRI, SDRR, total power are all measures of global HRV, and higher values indicate a more adaptable cardiac autonomic function [4]. Fourteen studies used one of these variables to determine global HRV before and after OSA treatment. Figure 2 shows there was no significant overall effect of OSA treatment on global HRV (Hedges’ g = 0.02, 95% CI −0.32, 0.36). There was considerable heterogeneity between studies (*Q* = 92.18, *df* = 18, *p* < .001; *I*^2^ = 80.47%). Symmetry in the funnel plot (Supplementary Figure S2) and Egger’s test suggests no significant publication bias (*p* = .27).

### (ii) Sympathetic HRV

**Figure 3.**
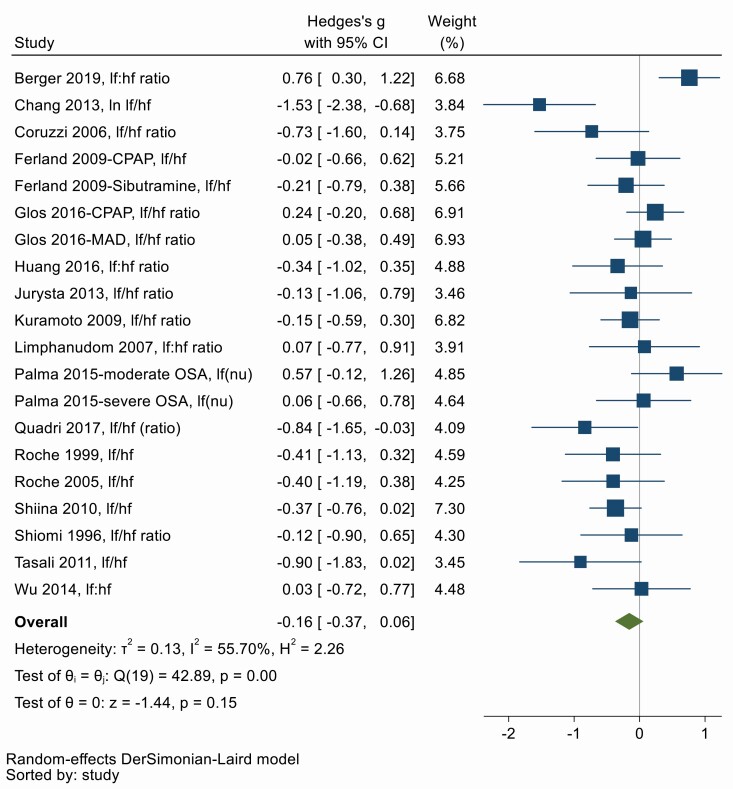
Forest plot for effect of OSA treatment on sympathetic measures of heart rate variability (*n* = 14 studies).

Low frequency to high frequency (LF:HF) ratio of HRV is a frequency domain measure of cardiac sympathovagal balance and an elevated LF:HF ratio suggests sympathetic predominance [25], similarly SD1:SD2 ratio is a nonlinear measure of HRV and is correlated with the LF:HF ratio [26]. The low frequency (LF) component when expressed in normalized units (nu) is regard as a marker of sympathetic modulation [25]. Therefore, elevated measures of LF:HF, SD1:SD2, and LF(nu) suggests sympathetic predominance. 17 studies used one of these variables to determine sympathetic modulation of HRV before and after OSA treatment. Figure 3 shows there was a tendency towards a small decrease in sympathetic measures of HRV with OSA treatment, although this was not statistically significant (Hedges’ *g* = −0.16, 95% CI −0.37, 0.06). There was moderate heterogeneity between studies (*Q* = 42.89, *df* = 19, *p* < .001; *I*^2^ = 55.70%). Asymmetry in the funnel plot (Supplementary Figure S2) and Egger’s test suggests there is evidence of publication bias (*p* = .02). The trim-and-fill method suggests that the overall effect is zero once six missing studies and their effect studies are imputed and included (Hedges’ *g* = 0.04, 95% CI −0.19, 0.27). The limited meta-regression of *n* = 8 studies suggests treatment adherence may modify the overall effect of treatment, however, this is in the opposite direction than expected (β = 2.81; 95% CI −0.40–6.02; *p* = .09), possibly indicating that better controlled and better reported studies generally report smaller effect sizes. There were no significant effects of other treatment or patient characteristics (Appendix 5).

### (iii) Parasympathetic HRV

**Figure 4.**
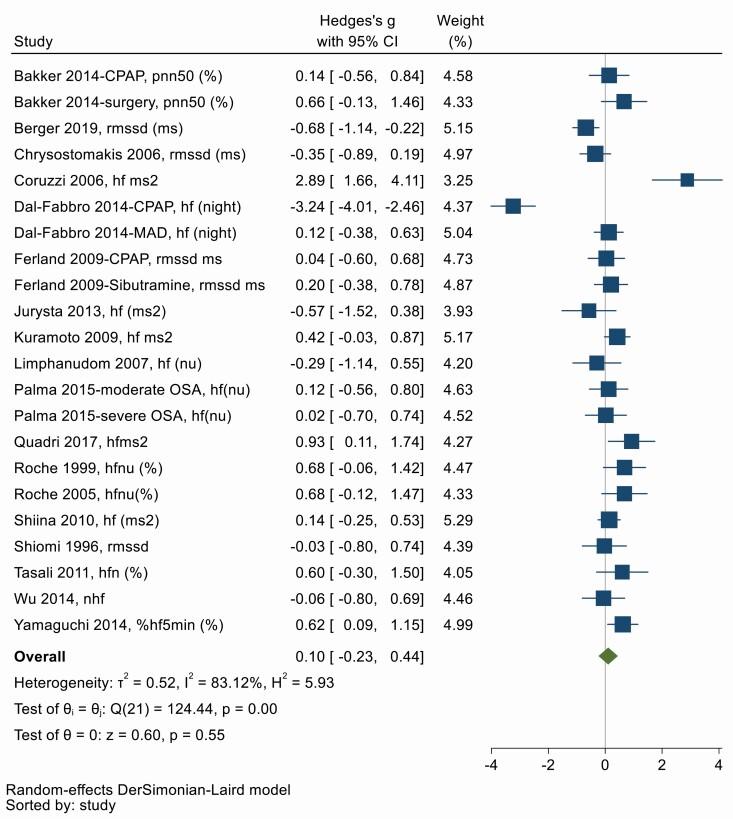
Forest plot for effect of OSA treatment on parasympathetic measures of heart rate variability (*n* = 19 studies).

RMSSD, pNN50, NN50, HF (ms^2^), HF (n.u), SD1 are all measures of vagal modulation of the heart and higher values indicate parasympathetic predominance [25, 27]. Nineteen studies used one of these variables to determine parasympathetic modulation of HRV before and after OSA treatment. Figure 4 shows there was no overall change in parasympathetic measures of HRV with OSA treatment (Hedges’ *g* = 0.10, 95% CI −0.23, 0.44). There was significant heterogeneity between studies (*Q* = 124.44, *df* = 21, *p* < .001; *I*^2^ = 83.12%). Symmetry in the funnel plot (Supplementary Figure S2) and Egger’s test suggests no significant publication bias (*p* = .16).

### (iv) Low frequency HRV and SD2

**Figure 5.**
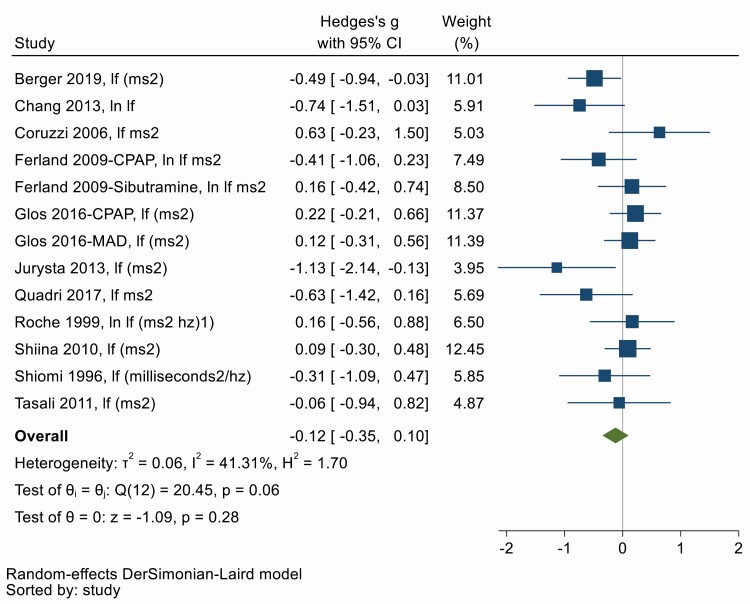
Forest plot for effect of OSA treatment on low frequency heart rate variability (*n* = 11 studies).

The interpretation of the Low frequency (LF) component is controversial, and it is regarded as a marker that includes both sympathetic and vagal influences [25]. Similarly, SD2, a non-linear measure of HRV, is highly correlated with the LF. Thirteen studies measured LF or SD1 to determine both sympathetic and parasympathetic modulation of HRV before and after OSA treatment. Figure 5 shows there was no change in low-frequency measures of HRV with OSA treatment (Hedges’ *g* = −0.12, 95% CI −0.35, 0.10). There was low to moderate heterogeneity between studies (*Q* = 20.15, *df* = 12, *p* = .06; *I*^2^ = 41.31%). Symmetry in the funnel plot (Supplementary Figure S2) and Egger’s test suggests no significant publication bias (*p* = 0.16). Univariate meta-regression provides evidence for an impact of treatment duration, that is, increasing duration of treatment is associated with larger decrements in HRV (β = −0.29; 95% CI −0.58, −0.01, *p* = .04). Treatment adherence, baseline OSA, patient age, and sex did not appear to influence the effect of treatment. However, this must be cautiously interpreted due to the small number of studies and the fact that only univariate regression was possible (Appendix 5).

### (v) Very low frequency HRV

**Figure 6.**
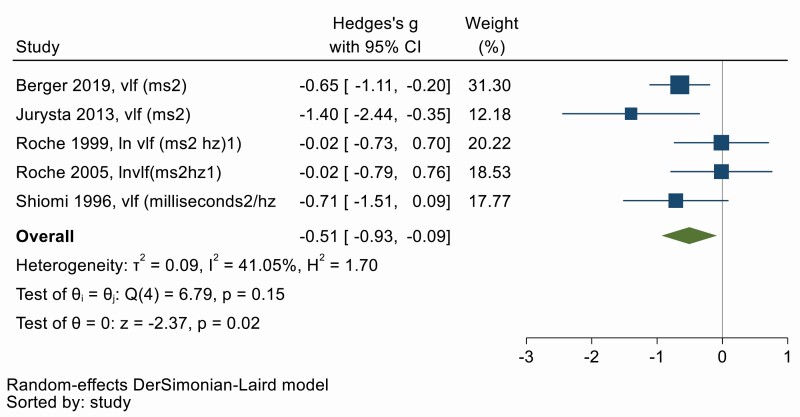
Forest plot for effect of OSA treatment on very low frequency heart rate variability (*n* = 5 studies).

The physiological correlates of Very low frequency (VLF) of HRV are unknown. Five studies evaluated VLF before and after OSA treatment [25]. VLF HRV decreased with OSA treatment as shown in Figure 6, with a moderate effect size (Hedges’ *g* = −0.51, 95% CI −0.93, −0.09). There was low heterogeneity between studies (*Q* = 6.79, *df* = 4, *p* = 0.15; *I*^2^ = 41.05%). Symmetry in the funnel plot (Supplementary Figure S2) and Egger’s test shows no evidence of publication bias (*p* = .56). Univariate meta-regression showed that increasing treatment duration is correlated with a larger decrease in VLF HRV (β = −0.31; 95% CI −0.61, −0.02, *p* = .04) and that older age appears to reduce the benefit of treatment (β = 0.07, 95% CI 0.00, 0.15, *p* = .05). However, these results will need to be replicated as more primary studies become available.

### Baroreceptor function

**Figure 7.**
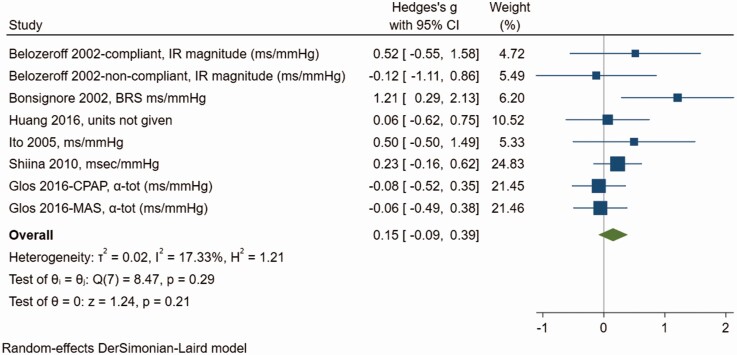
Forest plot for effect of OSA treatment on baroreceptor function (*n* = 6 studies).

BRS, sBRS, and baroreflex gain, are all measures of baroreceptor function. Low baroreceptor function or sensitivity is an unfavorable prognostic marker of autonomic dysfunction. There were six studies investigating the impact of OSA treatment on baroreceptor function with two studies each contributing two comparisons between patients treated with OSA and MAS and patients who were adherent to CPAP and those who were not, resulting in eight comparisons in total. Meta-analysis demonstrated no significant effect of treatment on baroreceptor function (Figure 7), although the effect size tended towards an increase (Hedges’ *g* = 0.15, 95% CI −0.09, 0.39). There was little heterogeneity between studies (*Q* = 8.47, *df* = 7, *p* = .29; *I*^2^ = 17.33%). Egger’s test suggests no significant publication bias (*p* = .27) although there is some asymmetry in the funnel plot (Supplementary Figure S3). Trim-and-fill imputed two missing studies on the left of the plot, although inclusion of these studies would be consistent with no significant overall effect of treatment on baroreceptor function (0.06; −0.23, 0.35). Univariate meta-regressions shows that more severe OSA is correlated with a larger effect of treatment (β = 0.01; 95% CI 0.00, 0.03, *p* = .04) and a larger proportion of men in the study was also associated with a larger treatment effect (β = 3.70; 95% CI 0.31, 7.06, *p* = .03). On multivariate meta-regression, the effect seems attributable mostly to sex differences, but this is based only on seven studies and does not reach statistical significance (Appendix 5).

### Catecholamines

**Figure 8.**
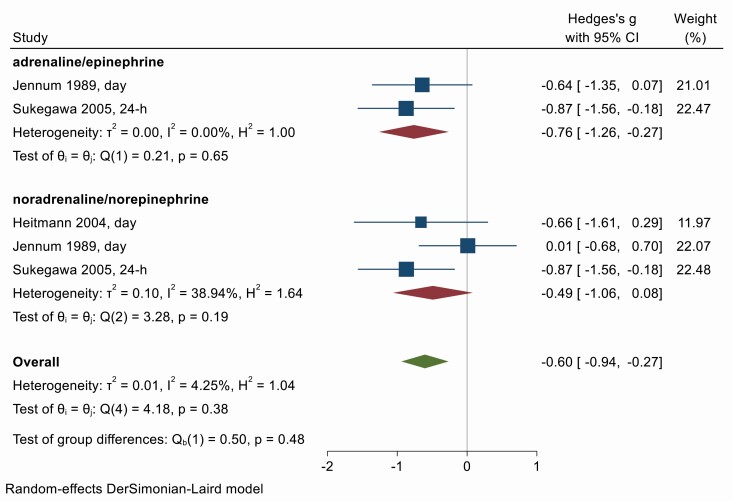
Forest plot for effect of OSA treatment on catecholamines (*n* = 3 studies).

Circulating catecholamines are both a hormone and a postganglionic sympathetic nervous system mediator modulated by the autonomic nervous systems, and elevated levels are associated with sympathoexcitation [28]. There were only three studies investigating the impact of OSA treatment on catecholamines, specifically adrenaline (epinephrine) and noradrenaline (norepinephrine) (Figure 8). There was a large reduction in catecholamines with treatment (Hedges g = −0.60, 95% CI −0.94, −0.27) and little heterogeneity between studies (*Q* = 4.18, *df* = 4, *p* = .38; *I*^2^ = 4.25%). Egger’s test suggests no significant publication bias (*p* = .94) although there were fewer than 10 studies in the analysis. Trim-and-fill suggests one missing study on the right of the funnel plot (Supplementary Figure S4), the inclusion of which reduces the overall effect slightly (−0.53; −0.83, −0.22). Time of day may play a role as Heitmann 2004 suggests the effect on 24-h catecholamines is driven mainly by changes in daytime levels, with no change in nighttime levels. Meta-regression was not attempted due to the small number of studies involved.

### Muscle sympathetic nerve activity

**Figure 9.**
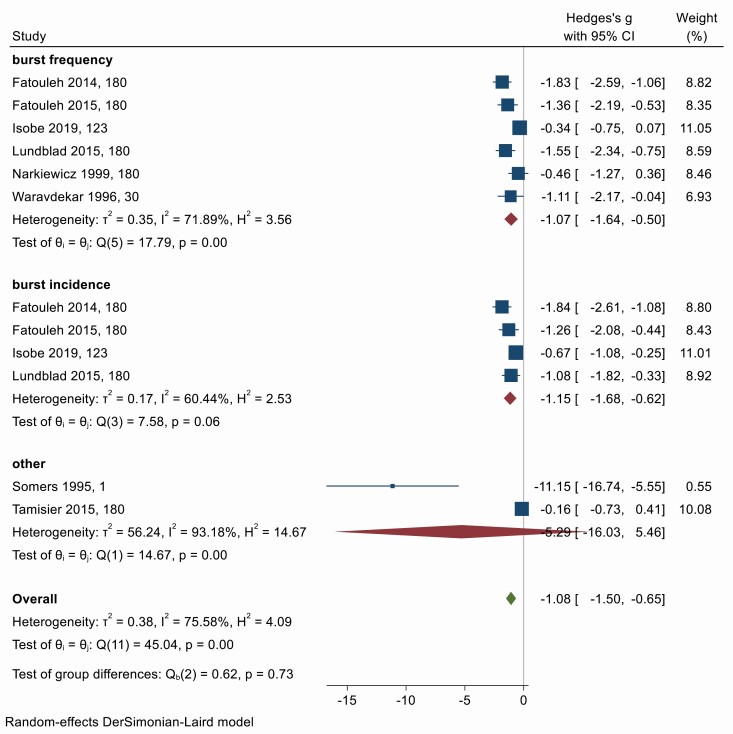
Forest plot for effect of OSA treatment on measures of muscle sympathetic nerve activity (*n* = 8 studies).

Muscle sympathetic nerve activity (MSNA) is measured using microneurography, an electrophysiological technique used for recording sympathetic nerve traffic directly from the human peripheral nerves. There were eight studies investigating the impact of OSA treatment on measures of MSNA. Large reductions in burst frequency (Hedges’ *g* = −1.07; 95% CI −1.64, −0.50), burst incidence (−1.15; −1.68, −0.62), and other measures of MSNA (−1.15; −1.68, −0.62) were observed (Figure 9). There was evidence of significant heterogeneity (overall *Q* = 45.04, *df* = 11, *p* = 0.00; *I*^2^ = 75.58%). Asymmetry in the funnel plot (Supplementary Figure S5) and Egger’s test shows evidence of small-study effects or publication bias (*p* < .001). Trim-and-fill imputation of two missing studies decreases the effect size only slightly to −0.94 (95% CI −1.40, −0.49). Meta-regression shows that the effect of treatment was modified by treatment adherence and sex (Appendix 5). On multivariate analysis including treatment adherence, % male patients, mean patient age, and baseline OSA severity, there was a trend towards greater treatment adherence being associated with larger effects of treatment (β = −8.50, 95% CI −18.05, 1.04, *p* = .08). The benefit of treatment is smaller in studies with a larger proportion of men (β = 11.63, 95% CI 2.95, 20.31, *p* = .01).

### MIBG (radionuclide imaging)

**Figure 10.**
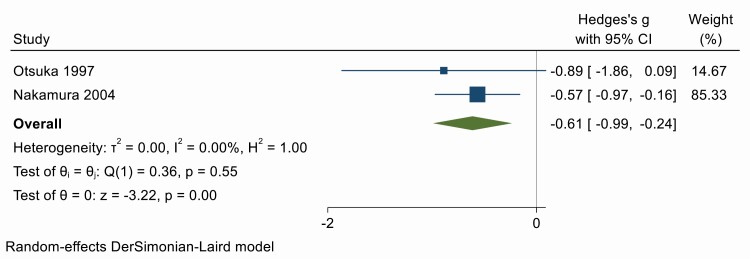
Forest plot for effect of OSA treatment on MIBG (*n* = 2 studies).

There were only two studies investigating the impact of OSA treatment on MIBG, specifically the washout rate (%), an index of sympathetic tone [29]. Meta-analysis demonstrated a moderate reduction in washout rate with treatment (Hedges’ *g* = −0.61; 95% CI −0.99, −0.24) (Figure 10). There was little heterogeneity between the two studies (*Q* = 0.36, *df* = 1, *p* = .55; *I*^2^ = 0%). Egger’s test provided no evidence of publication bias (*p* = .55) however, trim-and-fill imputation of one missing study to balance the funnel plot (Supplementary Figure S6) does not substantially reduce the estimated effect (−0.57; −0.92, −0.22). Meta-regression was not attempted.

## Discussion

This meta-analysis examined the effect of OSA therapy on autonomic function, as assessed by HRV, baroreflex function, catecholamines, MSNA, and MIBG. Our results found improvements in catecholamines, MSNA, and MIBG measures of ANS function. However, there was no effect of OSA therapy on HRV and baroreflex measures of ANS function.

A large number of studies show a clear association between OSA and CVD prevalence and epidemiological studies show that OSA is associated with increased incidences of CVD [2]. However, three randomized controlled trials (RCT) tested the effect of CPAP on cardiovascular risk reduction for OSA patients, and all showed no improvement in cardiovascular end points [30]. We believe a vitally important step is to study intermediatory mechanisms of cardiovascular risk to establish mechanistic plausibility of the treatment. As such, assessment of autonomic function has played an important role in understanding the disease pathology in both clinical and research settings and increased sympathetic activity is a hallmark of hypertension, major risk factor for CVD. It is widely hypothesized that OSA therapy reduces sympathetic overactivity and improves parasympathetic activity, restoring the impaired sympathovagal balance in OSA patients, and that this is a potential mechanism reducing the risk of cardiovascular diseases. However, the impact of OSA therapy on autonomic function is debated and clear mechanisms are unknown.

HRV is a noninvasive measure of cardiac autonomic control and was the most common method utilized to assess autonomic function. It is the analysis of the beat-to-beat variability in heart rate modified by the sympathetic and parasympathetic nervous system [25]. Higher measures of global HRV are indicative of a more adaptable cardiac autonomic function and reduced global measures of HRV are associated with increased morbidity and mortality [25]. The majority of reviewed studies assessed global measures of HRV, however there was no significant effect of OSA treatment on global HRV. The lack of significant effects despite a large number of studies on HRV is likely due to heterogeneity between studies, particularly the variation in the specific index of global HRV used in each study, combined with variation in the results of each study due to the sensitivity of HRV to external influences. Time domain analysis of HRV used to assess global HRV is strongly influenced by the condition and length of recording [25]. The most common method of assessment of HRV in OSA patients is during sleep due to the easily accessible ECG signal during sleep studies. However, assessment of global HRV during sleep, especially in patients with OSA with disordered respiratory rhythm will minimize the sensitivity of HRV as a tool for assessing the efficacy of OSA therapy on global measures of HRV [31]. Long duration of data collection utilizing 24-h Holter monitoring to assess SDNN as a marker of global HRV show the strongest association with cardiovascular risk and favor HRV assessment, accounting for metabolic and circadian variability [31]. Future studies in the sleep field, utilizing HRV as a tool to assess autonomic function should look to standardize measurements over longer duration of data collection.

Our results indicate statistically significant improvements in three out of the four measures of sympathetic function, namely Frequency domain analysis of HRV (LF:HF ratio), catecholamine, MSNA and radio nucleotide imaging. Specifically, OSA treatment moderately decreased catecholamines and MIBG but these results were based on few studies and require replication. OSA treatment led to a large decrease in indices of MSNA and there was a tendency towards a small decrease in the LF:HF ratio of HRV with OSA treatment, although this was not statistically significant. A recent meta-analysis showed CPAP therapy led to a small reduction in LF:HF ratio of HRV during sleep [32]. A number of possible pathophysiologic mechanisms may explain the relationship between OSA therapy and sympathetic function. It is hypothesized that ameliorating hypoxia and hypercapnia may diminish the cyclical pattern of heart rate and blood pressure surges through decreased chemoreflex sensitivity and increased baroreflex sensitivity, further reducing circulating catecholamines [33], collectively decreasing sympathetic activity and improving parasympathetic activity. Results from our meta-analysis demonstrated no significant effect of OSA treatment on baroreflex function, although the effect size tended towards an increase in baroreflex function and more research in this field is needed. Furthermore, parasympathetic function assessed by time and frequency domain measures of HRV was unchanged with OSA therapy. Similarly, a recent meta-analysis showed CPAP therapy had no effect on the high frequency component on of HRV during sleep [33]. Despite a large number of studies, we found a lack of significant effects in parasympathetic function in our meta-analysis. This may be explained by heterogeneity between studies and poor control of the conditions under which HRV was assessed.

A potential source of heterogeneity in autonomic response to therapy is variations in the pathophysiological causes of OSA within specific studies. A range of non-anatomical pathophysiological endotypes have been recognized, including low arousal threshold, poor muscle responsiveness, and high loop gain [34, 35] An unstable respiratory control system is one of the major non-anatomical mechanisms and can be quantified using loop gain (LG). LG or responsiveness is the theoretical product of the chemoreceptors. The carotid chemoreceptors detect oxygen in the body, exerting a reflex-mediated increase in ventilation and powerful stimulation of sympathetic vasoconstrictor outflow to the skeletal muscle, renal and mesenteric vascular beds. Respectively, CPAP is effective in ameliorating hypoxia, thus lowering LG and consequently MSNA, in part explaining the reduction of MSNA in a number of studies in this review. A low arousal threshold in OSA is thought to be another key driver of hemodynamic instability, contributing to changes in HRV. The arousal threshold can be manipulated pharmacologically with caution, using sedative and hypnotic agents such as trazodone and eszopiclone, however, studies show lack of a significant effect on the arousal threshold in response to CPAP [36], consequently, explaining the marginal changes seen in HRV with CPAP, in this review. Furthermore, the impact of OSA treatment on the various perturbations of OSA (intermittent hypoxia, sleep fragmentation, and intrathoracic pressure swings) may vary from patient to patient, producing variation in autonomic responses.

Adherence to OSA therapy is another important factor when considering effects of OSA therapy on cardiovascular function and outcomes. Sub-analysis of the randomized intervention with CPAP in coronary artery disease and OSA trial showed a cardiovascular risk reduction in patients who used CPAP for ≥ 4 h/night [37]. Although we planned to examine the influence of treatment adherence on autonomic function, there was insufficient data on treatment adherence for this to be carried out, especially given already small numbers of studies for the primary analysis, a limitation in this review. Similarly, our ability to examine the impact of mode of therapy, duration of therapy, baseline OSA severity, and patient demographics was limited and this will need to be explored in future reviews when more primary studies with full information on treatment and patient characteristics become available. The meta-regression analysis found some evidence of a dose–response effect of treatment, most reliably for MSNA, and potential sex differences in the impact of treatment. However, these results need to be interpreted with caution since all-together less than half of the reviewed studies were able to be included in meta-regression.

## Conclusion

The location of the autonomic nervous system renders it inaccessible to easy acquisition of direct physiological testing, thus relying on noninvasive, indirect measures. In this review, the strongest evidence for the effect of OSA therapy on autonomic function is seen in reduced sympathetic activity, as assessed by direct measures of MSNA, however, we did not see the expected improvement in parasympathetic function, assessed by HRV. Reduced sympathetic activity maybe one mechanism by which OSA therapy may reduce the risk of cardiovascular disease in OSA. Future studies with standardized protocols for noninvasive assessment of autonomic function are needed and blood biomarkers of sympathetic activation may enable a better understanding of anatomic function and its role in cardiovascular risk and approaches to therapy with OSA.

## Supplementary Material

zsac210_suppl_Supplementary_Material
