# Supplementary material for: The effect of obstructive sleep apnea therapy on cardiovascular autonomic function: a systematic review and meta-analysis
Source: Sleep. 2022 Sep 15;45(12):zsac210. doi: 10.1093/sleep/zsac210 (PMC9742902; doi:10.1093/sleep/zsac210)
Supplement: zsac210_suppl_Supplementary_Material [file zsac210_suppl_supplementary_material.docx]

**TITLE**

**The Effect of Obstructive Sleep Apnea Therapy on Cardiovascular Autonomic Function: A Systematic Review and Meta-Analysis**

**AUTHORS**

Hasthi U. Dissanayake ^a, b^, Yu Sun Bin ^a, b^, Kate Sutherland ^a, b, c^, Seren Ucak ^a, b^, Philip de Chazal ^a, d^, Peter A. Cistulli ^a, b, c^

**AFFILIATIONS**

a Sleep Research Group, Charles Perkins Centre, The University of Sydney, Australia

b Northern Clinical School, Faculty of Medicine and Health, The University of Sydney, Australia

c Centre for Sleep Health & Research, Department of Respiratory Medicine, Royal North Shore Hospital, Australia

d School of Biomedical Engineering, University of Sydney, Sydney, NSW, 2006, Australia

**CORRESPONDING AUTHOR**

Hasthi Dissanayake

3E67 Sleep Research Group

D17 Charles Perkins Centre

University of Sydney NSW Australia 2006

[hasthi.dissanayake@sydney.edu.au](mailto:hasthi.dissanayake@sydney.edu.au)

**SUPPLEMENTARY MATERIALS**

Appendix 1. Definitions and indices of different aspects of autonomic function and typical associations with health outcomes.

Appendix 2. Search strategy

Appendix 3. Risk of bias assessment.

Appendix 4. Funnel plots for each autonomic function outcome.

Appendix 5. Results of meta-regression on outcomes with available data.

| **Appendix 1. Definitions and indices of different aspects of autonomic function and typical associations with health outcomes.** | | | |
| --- | --- | --- | --- |
| **Aspect of autonomic function** | **Specific index of measures** | **Definition** | **Health outcome** |
| Heart Rate Variability (HRV) | CoV(%), CVRR, mean RR, mean NN, NN interval, RRI, RR interval variation, RR variability, SD, SDNN, SDaNN, SDIndex, SDRI, SDRR, total power, | Global HRV  ↑ values indicate an adaptable ANS | + |
|  | RMSSD, pNN50, NN50, HF (ms^2^), HF (n.u), SD1 | ↑ parasympathetic predominance | + |
|  | LF:HF ratio, LF (nu), SD1:SD2 ratio | ↑ sympathetic predominance | - |
|  | LF, SD2 | Physiological correlates debated:   1. Index of sympathetic branch 2. Modern research prefer to view it as both sympathetic and parasympathetic modulation | 1. - 2. + |
|  | VLF, Ln VLF | Physiological correlates still unknown. | ? |
| Baroreceptor function | BRS, sBRS, baroreflex gain | ↑ Baroreceptor function | + |
| Catecholamines | Norepinephrine (norepinephrine) | ↑ sympathetic response | - |
|  | Adrenaline (epinephrine) |  |  |
| Muscle Sympathetic Nerve activity (MSNA) | ↑ Burst frequency  ↑ Burst incidence | ↑ sympathetic response | - |
| Radionuclide imaging | Sympathetic neurotransmitter uptake and clearance in the heart | ↑ Myocardial sympathetic function | - |
| ↑increase ; ↓decrease; + positive health outcome; - negative health outcome.  CoV(%) = coefficient of variation (percent); CVRR = coefficient of variation of the R-R interval; RRI = R-R interval; SD = standard deviation; SDNN = standard deviation of the N-N interval; SDRR = standard deviation of the R-R interval SDANN=standard deviation of 5-min average NN intervals; SDRI (SDRI index) = mean of the standard deviation of all RR (NN) intervals for all 5-min segments of the entire recording; RMSSD=root mean squared successive difference; PNN50=percentage of adjacent NN intervals; the NN50 count divided by the total number of all RR intervals; HF=high frequency; HF (n.u)=normalized units of high frequency component; SD1 = Poincare plot standard deviation perpendicular the line of identity; SD2 = Poincare plot standard deviation along the line of identity; LF:HF=ratio of low frequency and high frequency; LF (n.u)=normalized units of low frequency component; SD1:SD2 = ratio of SD1 to SD2; LF=low frequency; VLF= very low frequency  BRS = baroreceptor sensitivity, sBRS = spontaneous baroreceptor sensitivity. | | | |

**Appendix 2. Search strategy**

MEDLINE

| 1. Sleep Apnea, Obstructive/ |
| --- |
| 2. ("sleep apn?ea hypopnea syndrome" or sahs).mp. |
| 3. ((apn?ea or apneic or "disordered breathing") adj4 (sleep or nocturnal)).mp. |
| 4. ("obstructive sleep apn?ea hypopnea syndrome" or osahs).mp. |
| 5. ("obstructive sleep apn?ea" or osa or osas).mp. |
| 6. 1 or 2 or 3 or 4 or 5 |
| 7. exp Autonomic Nervous System/ |
| 8. ((autonomic or sympathetic or parasympathetic) adj3 "nervous system").mp. |
| 9. 7 or 8 |
| 10. 6 and 9 |
| 11. exp Electrocardiography/ |
| 12. Heart Rate/ |
| 13. Baroreflex/ |
| 14. Pressoreceptors/ |
| 15. Blood Pressure/ |
| 16. (electrocardiography or "heart rate" or baroreflex or baroreceptor* or "blood pressure" or "cardiac autonomic control" or "autonomic dysfunction" or "muscle sympathetic nerve activity" or MSNA).mp. |
| 17. 11 or 12 or 13 or 14 or 15 or 16 |
| 18. exp Adult/ |
| 19. adult*.mp. |
| 20. 18 or 19 |
| 21. 10 and 17 and 20 |
| 22. limit 21 to human |

EMBASE

| 1. exp sleep disordered breathing/ |
| --- |
| 2. ("sleep apn?ea hypopnea syndrome" or sahs).mp. |
| 3. ((apn?ea or apneic or "disordered breathing") adj4 (sleep or nocturnal)).mp. |
| 4. ("obstructive sleep apn?ea hypopnea syndrome" or osahs).mp. |
| 5. ("obstructive sleep apn?ea" or osa or osas).mp. |
| 6. 1 or 2 or 3 or 4 or 5 |
| 7. exp Autonomic Nervous System/ |
| 8. ((autonomic or sympathetic or parasympathetic) adj3 "nervous system").mp. |
| 9. 7 or 8 |
| 10. 6 and 9 |
| 11. exp Electrocardiography/ |
| 12. exp Heart Rate/ |
| 13. pressoreceptor reflex/ |
| 14. exp pressoreceptor/ |
| 15. exp blood pressure/ |
| 16. (electrocardiography or "heart rate" or baroreflex or baroreceptor* or "blood pressure !" or "cardiac autonomic control" or "autonomic dysfunction" or "muscle sympathetic nerve activity" or MSNA).mp. |
| 17. 11 or 12 or 13 or 14 or 15 or 16 |
| 18. exp Adult/ |
| 19. adult*.mp. |
| 20. 18 or 19 |
| 21. 10 and 17 and 20 |
| 22. limit 21 to human |

3. SCOPUS

( TITLE-ABS-KEY ( "autonomic nervous system" OR "sympathetic nervous system" OR "parasympathetic nervous system" OR "electrocardiography" OR "heart rate" OR "baroreflex" OR "heart rate" OR "muscle sympathetic nerve activity" ) ) AND ( TITLE-ABS-KEY ( "obstructive sleep apnea" OR "sleep disordered breathing" ) ) AND ( TITLE-ABS-KEY ( "adult" ) ) AND ( LIMIT-TO ( EXACTKEYWORD , "Humans" ) )

**Appendix 3. Risk of bias assessment.**

**Figure S1a. Risk of bias graph.**

**Figure S1b. Risk of bias summary.**

| **Author** | **Random Sequence generation** | **Allocation concealment** | **Blinding of participants and personnel** | **Blinding of outcome assessment** | **Incomplete outcome data** | **Selective reporting** |
| --- | --- | --- | --- | --- | --- | --- |
| Bakker, et al. 2014 | **-** | **-** | **-** | **+** | **+** | **+** |
| Belozeroff, et al. 2002 | **-** | **-** | **-** | **+** | **+** | **+** |
| Berger, et al. 2019 | **+** | **?** | **+** | **+** | **+** | **+** |
| Bonsignore, et al. 2002 | **-** | **-** | **+** | **+** | **+** | **+** |
| Chang, et al. 2013 | **-** | **-** | **+** | **+** | **+** | **+** |
| Chrysostomakis, et al. 2006 | **-** | **-** | **+** | **+** | **+** | **+** |
| Coruzzi, et al. 2006 | **-** | **-** | **+** | **+** | **+** | **+** |
| Dal-Fabbro, et al. 2014 | **+** | **+** | **+** | **+** | **-** | **+** |
| Fatouleh, et al. 2014 | **-** | **-** | **+** | **+** | **+** | **+** |
| Fatouleh, et al. 2015 | **-** | **-** | **+** | **+** | **-** | **+** |
| Ferland, et al. 2009 | **-** | **-** | **+** | **+** | **+** | **+** |
| Glos, et al. 2016 | **+** | **?** | **+** | **+** | **-** | **+** |
| Heitmann, et al. 2004 | **-** | **-** | **+** | **+** | **-** | **+** |
| Huang, et al. 2016 | **-** | **-** | **+** | **+** | **+** | **+** |
| Isobe, et al. 2019 | **-** | **-** | **+** | **+** | **-** | **+** |
| Ito, et al. 2005 | **-** | **-** | **+** | **+** | **+** | **+** |
| Jennum, et al. 1989 | **-** | **-** | **+** | **+** | **+** | **+** |
| Jurysta, et al. 2013 | **-** | **-** | **+** | **+** | **+** | **+** |
| Kim, et al. 2020 | **-** | **-** | **+** | **+** | **+** | **+** |
| Kufoy, et al. 2012 | **-** | **-** | **+** | **+** | **+** | **+** |
| Kuramoto, et al. 2009 | **-** | **-** | **+** | **+** | **+** | **?** |
| Limphanudom, et al. 2007 | **-** | **-** | **+** | **+** | **?** | **+** |
| Marrone, et al. 1993 | **-** | **-** | **+** | **+** | **+** | **+** |
| Nakamura, et al. 2004 | **-** | **-** | **+** | **+** | **+** | **+** |
| Narkiewicz, et al. 1999 | **-** | **-** | **+** | **+** | **-** | **+** |
| Nelesen, et al. 2001 | **+** | **-** | **+** | **+** | **?** | **+** |
| Noda, et al. 2007 | **+** | **-** | **+** | **+** | **-** | **+** |
| Otsuka, et al. 1997 | **-** | **-** | **+** | **+** | **-** | **+** |
| Palma, et al. 2015 | **-** | **-** | **+** | **+** | **+** | **+** |
| Quadri, et al. 2017 | **-** | **-** | **+** | **+** | **+** | **+** |
| Roche, et al. 2005 | **-** | **-** | **+** | **+** | **+** | **+** |
| Roche, et al. 1999 | **-** | **-** | **+** | **+** | **+** | **+** |
| Schytz, et al. 2013 | **-** | **-** | **+** | **+** | **-** | **+** |
| Shiina, et al. 2010 | **-** | **-** | **+** | **+** | **+** | **+** |
| Shiomi, et al. 1996 | **-** | **-** | **+** | **+** | **+** | **+** |
| Somers, et al. 1995 | **-** | **-** | **+** | **+** | **-** | **+** |
| Sukegawa, et al. 2005 | **-** | **-** | **+** | **+** | **?** | **+** |
| Tamisier, et al. 2015 | **-** | **-** | **+** | **+** | **-** | **+** |
| Tasali, et al. 2011 | **-** | **-** | **+** | **+** | **+** | **+** |
| Waravdekar, et al. 1996 | **-** | **-** | **+** | **+** | **+** | **+** |
| Wu, et al. 2014 | **-** | **-** | **+** | **+** | **+** | **+** |
| Yamaguchi, et al. 2014 | **-** | **-** | **+** | **+** | **+** | **+** |
| Ziegler, et al. 2001 | **+** | **+** | **+** | **+** | **-** | **+** |

**Appendix 4. Funnel plots for each autonomic function outcome.**

**Figure S2. Funnel plots for each category of HRV outcome**

**Figure S3. Funnel plot for baroreceptor function**

**Figure S4. Funnel plot for catecholamines**

**Figure S5. Funnel plot for studies of MSNA**

**Figure S6. Funnel plot for studies of MIBG**

**Appendix 5. Results of meta-regression on outcomes with available data**

Variables extracted for meta-regression were:

- **Treatment**: CPAP or other (MAS, surgery, sibutramine)
- **Treatment duration:** coded as 1 to n days (e.g. 365 days = 1 year). Where treatment duration was reported in studies as a range (e.g. 12 to 18 months), we substituted a midpoint (e.g. 15 months = 450 days)
- **Treatment adherence**: coded as 0 to 100%. Outcomes measured during treatment were entered as 100% adherence, 50% adherence = 4 hours per night. If the study reported e.g. use of treatment for 4 hours per night for 70% of nights during the study, then treatment adherence = 4 x 0.7 = 35%.
- **OSA severity:** coded as mean AHI/RDI at baseline.
- **Sex:** the proportion of male patients in the study (0 to 100%).
- **Age:** the mean age of patients in the study (in years). Median was used if the mean was not reported.

**Data completeness for meta-regression:** As can be seen below, more than half of the studies for the majority of outcomes could not be included in meta-regression due to missing data.

|  |  | **Comparisons with data on:** | | | | | | |
| --- | --- | --- | --- | --- | --- | --- | --- | --- |
| **Outcome** | **Total no. comparisons*** | **Treatment**  **duration** | **Treatment  adherence** | **OSA  severity** | **Patient  sex** | **Patient  age** | **Full  data** |  |
| HRV - Global | 19  (12 PAP, 4 MAS, 3 other) | 19 | 7 | 19 | 19 | 19 | 7 |  |
| *Restricted to PAP only* | *12* | *12* | *6* | *12* | *12* | *12* | *6* |  |
| HRV - Sympathetic | 20  (14 PAP, 3 MAS, 3 other) | 20 | 11 | 20 | 18 | 19 | 9 |  |
| *Restricted to PAP only* | *14* | *14* | *9* | *14* | *13* | *14* | *7* |  |
| HRV - Parasympathetic | 22  (16 PAP, 3 MAS, 3 other) | 22 | 9 | 22 | 21 | 22 | 8 |  |
| *Restricted to PAP only* | *16* | *16* | *8* | *16* | *15* | *16* | *7* |  |
| HRV - Low frequency | 13 (8 PAP, 3 MAS, 2 other) | 13 | 5 | 13 | 13 | 13 | 5 |  |
| *Restricted to PAP only* | *8* | *8* | *4* | *8* | *8* | *8* | *4* |  |
| HRV - Very low frequency | 5 (3 PAP, 1 MAS, 1 other) | 5 | 2 | 5 | 5 | 5 | 2 |  |
| *Restricted to PAP only* | *3* | *3* | *2* | *3* | *3* | *3* | *2* |  |
| BRS | 8 (6 PAP, 1 MAS, 1 other) | 8 | 8 | 4 | **7** | **7** | 3^#^ |  |
| Catecholamines | 3 | 3 | 3 | 3 | 2 | 3 | 2 |  |
| MSNA | 12 | 12 | 9 | 10 | 11 | 12 | 8 |  |
| MIBG | 2 | 2 | 0 | 2 | 1 | 1 | 0 |  |

*Note: The number of comparisons differs from the number of studies. The fact that multiple comparisons came from the same studies contributed to large amounts of missing data and collinearity (#) when it came to attempting meta-regression.

**Results of univariate and multivariate meta-regression on outcomes with available data:** The following results should be interpreted with caution.

**HRV global (n=19 comparisons):** Treatment and patient characteristics did not modify the treatment effect.

| **Univariate (n=19 max)** | | | | | **Multivariable (n=7)** | | | | | |
| --- | --- | --- | --- | --- | --- | --- | --- | --- | --- | --- |
| **Variable** | **Coef** | **[95% Conf.** | **Interval]** | **P>z** | **Variable** | **Coef** | **[95% Conf.** | **Interval]** | **P>z** |  |
| Treatment |  |  |  |  | Treatment |  |  |  |  |  |
| MAS | -0.17 | -1.05 | 0.71 | 0.71 | MAS | - |  |  |  |  |
| Other | 0.54 | -0.44 | 1.52 | 0.28 | Other | 0.46 | -0.75 | 1.67 | 0.46 |  |
| Tx duration | 0.00 | 0.00 | 0.00 | 0.67 | Tx duration | 0.00 | 0.00 | 0.00 | 0.80 |  |
| Tx adherence | -0.15 | -1.19 | 0.88 | 0.77 | Tx adherence | 0.11 | -3.37 | 3.58 | 0.95 |  |
| Baseline OSA | 0.00 | -0.02 | 0.02 | 0.68 | Baseline OSA | 0.00 | -0.07 | 0.08 | 0.92 |  |
| % Male | -1.23 | -3.05 | 0.59 | 0.19 | % Male | 3.69 | -22.96 | 30.34 | 0.79 |  |
| Mean age | -0.00 | -0.14 | 0.13 | 0.97 | Mean age | 0.07 | -0.14 | 0.28 | 0.51 |  |

**HRV global, restricted to PAP studies only (n=12 comparisons):** Treatment and patient characteristics did not modify the treatment effect.

| **Univariate (n=12 max)** | | | | | **Multivariate (n=6)** | | | | | |
| --- | --- | --- | --- | --- | --- | --- | --- | --- | --- | --- |
| **Variable** | **Coef** | **[95% Conf.** | **Interval]** | **P>z** | | **Variable** | **Coef** | **[95% Conf.** | **Interval]** | **P>z** |
| Tx duration | 0.00 | 0.00 | 0.00 | 0.82 | | Tx duration | 0.00 | 0.00 | 0.00 | 0.80 |
| Tx adherence | -0.29 | -1.43 | 0.85 | 0.62 | | Tx adherence | 0.11 | -3.43 | 3.65 | 0.95 |
| Baseline OSA | 0.01 | -0.02 | 0.04 | 0.47 | | Baseline OSA | 0.00 | -0.07 | 0.08 | 0.92 |
| % Male | 1.74 | -2.35 | 5.82 | 0.41 | | % Male | 3.69 | -23.50 | 30.88 | 0.79 |
| Mean age | 0.08 | -1.11 | 0.28 | 0.40 | | Mean age | 0.07 | -0.14 | 0.28 | 0.51 |

**HRV sympathetic (n=20 comparisons):** There was a trend towards treatment adherence modifying the treatment effect, however, this was in the opposite direction to that expected.

| **Univariate (n=20 max)** | | | | | **Multivariate (n=9)** | | | | | |
| --- | --- | --- | --- | --- | --- | --- | --- | --- | --- | --- |
| **Variable** | **Coef** | **[95% Conf.** | **Interval]** | **P>z** | **Variable** | **Coef** | **[95% Conf.** | **Interval]** | **P>z** |  |
| Treatment |  |  |  |  | Treatment |  |  |  |  |  |
| MAS | 0.03 | -0.59 | 0.64 | 0.93 | MAS | - |  |  |  |  |
| Other | 0.36 | -0.23 | 0.94 | 0.24 | Other | -0.37 | -1.64 | 0.91 | 0.57 |  |
| Tx duration | 0.00 | -0.00 | 0.00 | 0.67 | Tx duration | 0.00 | 0.00 | 0.00 | 0.81 |  |
| **Tx adherence** | **1.80** | **0.42** | **3.17** | **0.01** | **Tx adherence** | **2.81** | **-0.40** | **6.02** | **0.09** |  |
| Baseline OSA | -0.01 | -0.03 | 0.00 | 0.09 | Baseline OSA | -0.02 | -0.07 | 0.03 | 0.46 |  |
| % Male | 0.04 | -0.90 | 1.62 | 0.58 | % Male | 3.07 | -1.86 | 8.00 | 0.22 |  |
| Mean age | 0.01 | -0.03 | 0.05 | 0.63 | Mean age | -0.08 | -0.29 | 0.12 | 0.41 |  |

**HRV sympathetic - restricted to PAP studies only (n=14 comparisons):** There was a trend towards treatment adherence modifying the treatment effect but this was the opposite to what is expected (i.e. greater adherence = smaller treatment effect). This may be due to better controlled, conducted, and reported studies having smaller treatment effects.

| **Univariate (n=14 max)** | | | | | **Multivariate (n=8)** | | | | |
| --- | --- | --- | --- | --- | --- | --- | --- | --- | --- |
| **Variable** | **Coef** | **[95% Conf.** | **Interval]** | **P>z** | **Variable** | **Coef** | **[95% Conf.** | **Interval]** | **P>z** |
| Tx duration | 0.00 | 0.0 | 0.00 | 0.87 | Tx duration | 0.00 | 0.00 | 0.00 | 0.81 |
| **Tx adherence** | **1.95** | **0.44** | **3.46** | **0.01** | Tx adherence | 2.81 | -0.40 | 6.02 | 0.09 |
| Baseline OSA | -0.01 | -0.03 | 0.01 | 0.22 | Baseline OSA | -0.02 | -0.07 | 0.03 | 0.46 |
| % Male | 0.53 | -0.73 | 1.79 | 0.41 | % Male | 3.07 | -1.86 | 8.00 | 0.22 |
| Mean age | 0.01 | -0.04 | 0.05 | 0.78 | Mean age | -0.08 | -0.29 | 0.12 | 0.41 |

**HRV parasympathetic (n=20 comparisons):** Treatment and patient characteristics were not significant modifiers of the treatment effect.

| **Univariate (n=20 max)** | | | | | **Multivariate (n=8)** | | | | |
| --- | --- | --- | --- | --- | --- | --- | --- | --- | --- |
| **Variable** | **Coef** | **[95% Conf.** | **Interval]** | **P>z** | **Variable** | **Coef** | **[95% Conf.** | **Interval]** | **P>z** |
| Treatment |  |  |  |  | Treatment |  |  |  |  |
| MAS | 0.79 | -0.28 | 1.87 | 0.15 | MAS | - |  |  |  |
| Other | 0.03 | -1.00 | 1.05 | 0.96 | Other | 0.26 | -0.70 | 1.22 | 0.60 |
| Tx duration | 0.00 | 0.00 | 0.00 | 0.44 | Tx duration | 0.00 | 0.00 | 0.00 | 0.39 |
| Tx adherence | 1.14 | -0.95 | 3.23 | 0.29 | Tx adherence | 2.54 | -4.70 | 9.79 | 0.49 |
| Baseline OSA | -0.01 | -0.04 | 0.01 | 0.28 | Baseline OSA | 0.01 | -0.03 | 0.05 | 0.68 |
| % Male | -1.00 | -2.53 | 0.54 | 0.21 | % Male | 0.32 | -5.14 | 5.77 | 0.91 |
| Mean age | 0.02 | -0.04 | 0.07 | 0.49 | Mean age | -0.06 | -0.33 | 0.21 | 0.67 |

**HRV parasympathetic - restricted to PAP studies only (n=16 comparisons):** Treatment and patient characteristics were not significant modifiers of the treatment effect.

| **Univariate (n=16 max)** | | | | | **Multivariate (n=7)** | | | | |
| --- | --- | --- | --- | --- | --- | --- | --- | --- | --- |
| **Variable** | **Coef** | **[95% Conf.** | **Interval]** | **P>z** | **Variable** | **Coef** | **[95% Conf.** | **Interval]** | **P>z** |
| Tx duration | -0.00 | -0.00 | 00.00 | 0.55 | Tx duration | 0.00 | 0.00 | 0.00 | 0.39 |
| Tx adherence | 1.15 | -0.94 | 3.24 | 0.28 | Tx adherence | 2.54 | -4.70 | 9.79 | 0.49 |
| Baseline OSA | 0.00 | -0.05 | 0.03 | 0.88 | Baseline OSA | 0.01 | -0.03 | 0.05 | 0.68 |
| % Male | -0.71 | -2.69 | 1.27 | 0.48 | % Male | 0.32 | -5.14 | 5.77 | 0.91 |
| Mean age | 0.04 | -0.02 | 0.09 | 0.20 | Mean age | -0.06 | -0.33 | 0.21 | 0.67 |

**HRV low frequency (n=13 comparisons):** Treatment duration appears to modify the treatment effect, i.e. the longer the duration of treatment, the larger the treatment effect. Note treatment duration has been rescaled here so that 1 unit increase = 1 additional year of treatment. However, this effect cannot be seen in the multivariate regression, likely due to the small number of studies included (n=5 rather than n=13).

| **Univariate (n=13 max)** | | | | | **Multivariate (n=5)** | | | | |
| --- | --- | --- | --- | --- | --- | --- | --- | --- | --- |
| **Variable** | **Coef** | **[95% Conf.** | **Interval]** | **P>z** | **Variable** | **Coef** | **[95% Conf.** | **Interval]** | **P>z** |
| Treatment |  |  |  |  | Treatment |  |  |  |  |
| MAS | 0.33 | -0.26 | 0.92 | 0.27 | MAS | - |  |  |  |
| Other | 0.00 | -0.62 | 0.63 | 0.99 | Other | 0.53 | -2.47 | 3.53 | 0.73 |
| Tx duration/365 | **-0.30** | **-0.57** | **-0.02** | **0.03** | Tx duration/365 | 0.00 | -0.00 | 0.00 | 0.87 |
| Tx adherence | 1.10 | -0.88 | 3.09 | 0.28 | Tx adherence | 0.28 | -8.77 | 9.32 | 0.95 |
| Baseline OSA | -0.02 | -0.03 | 0.00 | 0.15 | Baseline OSA | -0.02 | -0.17 | 0.14 | 0.85 |
| % Male | -0.29 | -1.40 | 0.83 | 0.62 | % Male | Omitted due to collinearity | | | |
| Mean age | -0.00 | -0.04 | 0.04 | 0.96 | Mean age | Omitted due to collinearity | | | |

**HRV low frequency - restricted to PAP studies only (n=8 comparisons):** That duration of treatment modifies the treatment effect (observed above) is driven by the PAP treatment studies. Note treatment duration has been rescaled here so that 1 unit increase = 1 additional year of treatment.

| **Univariate (n=8 max)** | | | | | **Multivariate (n=4)** | | | | | | |
| --- | --- | --- | --- | --- | --- | --- | --- | --- | --- | --- | --- |
| **Variable** | **Coef** | **[95% Conf.** | **Interval]** | **P>z** | **Variable** | **Coef** | **[95% Conf.** | **Interval]** | **P>z** |  |  |
| Tx duration/365 | **-0.29** | **-0.58** | **-0.01** | **0.04** | Tx duration/365 | -0.12 | -0.64 | .041 | 0.67 |  |  |
| Tx adherence | 0.67 | -1.13 | 2.47 | 0.47 | Tx adherence | 0.28 | -2.29 | 2.84 | 0.83 |  |  |
| Baseline OSA | -0.02 | -0.04 | 0.01 | 0.13 | Baseline OSA | -0.02 | -0.06 | 0.03 | 0.54 |  |  |
| % Male | -0.32 | -1.56 | 0.92 | 0.62 | % Male | Omitted due to collinearity | | | | |  |
| Mean age | 0.01 | -0.04 | 0.05 | 0.77 | Mean age | Omitted due to collinearity | | | | |  |

**HRV very low frequency (n=5 comparisons) -** due to only n=2 studies with full data, only univariate analysis was carried out. Longer duration of treatment was correlated with a larger effect of treatment. There was also a trend towards patient age weakly moderating the effect of treatment i.e. larger treatment effects were observed in older patients.

| **Univariate (n=5 max)** | | | | | |
| --- | --- | --- | --- | --- | --- |
| **Variable** | **Coef** | **[95% Conf.** | **Interval]** | **P>z** |  |
| Treatment |  |  |  |  |  |
| MAS | -0.32 | -1.87 | 1.24 | 0.69 |  |
| Other | -0.26 | -1.67 | 1.16 | 0.72 |  |
| Tx duration/365 | **-0.31** | **-0.61** | **-0.02** | **0.04** |  |
| Tx adherence | 5.53 | -4.82 | 15.88 | 0.30 |  |
| Baseline OSA | 0.00 | -0.03 | 0.03 | 0.83 |  |
| % Male | -0.52 | -5.20 | 4.16 | 0.83 |  |
| Mean age | **0.07** | **0.00** | **0.15** | **0.05** |  |

**Baroreflex (n=8 comparisons):** Although there are 8 comparisons made, the data derive from only 6 studies. Together with missing data on OSA severity at baseline, the small number of studies causes collinearity between key variables, preventing multivariate (meta-)regression. If treatment adherence is omitted (given that treatment type differs in these studies - 6 CPAP, 1 MAS, 1 other), then univariately, male patients and baseline OSA severity moderate the treatment effect (i.e. both characteristics are correlated with bigger effect of treatment). However, this disappears on multiple regression.

| **Univariate (n=8 max)** | | | | | **Multivariate (n=7)** | | | | |
| --- | --- | --- | --- | --- | --- | --- | --- | --- | --- |
| **Effect** | **Coef.** | **[95% Conf.** | **Interval]** | **P>z** | **Effect** | **Coef.** | **[95% Conf.** | **Interval]** | **P>z** |
| Treatment (CPAP) |  |  |  |  | Treatment (CPAP) |  |  |  |  |
| MAS | -0.32 | -1.03 | 0.40 | 0.39 | MAS | 0.15 | -1.80 | 2.10 | 0.88 |
| Other | -0.20 | -1.09 | 0.69 | 0.67 | Other | - | - | - | - |
| Tx duration | 0.00 | -0.00 | 0.01 | 0.14 | Tx duration | 0.01 | -0.05 | 0.07 | 0.71 |
| Tx adherence* | - | - | - | - | Tx adherence* | - | - | - | - |
| Baseline OSA | **0.01** | **0.00** | **0.03** | **0.04** | Baseline OSA | -0.02 | -0.14 | 0.10 | 0.74 |
| % Male | **3.70** | **0.31** | **7.06** | **0.03** | % Male | 5.61 | -14.57 | 25.80 | 0.59 |
| Mean age | -0.02 | -0.13 | 0.09 | 0.74 | Mean age | 0.16 | -0.48 | 0.79 | 0.63 |

*Treatment adherence omitted due to missing data for other treatment modalities, causing collinearity.

**Catecholamines (n=5 comparisons):** Although there were 5 comparisons for the meta-analysis, this data only came from 3 studies and full data was only available on 2 of these studies so meta-regression was not attempted.

**MSNA (n=12 comparisons):** Multivariate meta-regression based on 8/12 comparisons indicates that the effect of treatment is reduced with more male patients and there is a trend towards treatment adherence enhancing the effect of treatment (i.e. decreasing MSNA). Note that the multivariate regression omits treatment duration due to collinearity.

| **Univariate (n=12 max)** | | | | | **Multivariate (n=8)** | | | | |
| --- | --- | --- | --- | --- | --- | --- | --- | --- | --- |
| **Effect** | **Coef.** | **[95% Conf.** | **Interval]** | **P>z** | **Effect** | **Coef.** | **[95% Conf.** | **Interval]** | **P>z** |
| Tx duration | 0.00 | -0.01 | 0.01 | 0.72 | Tx duration | - | - | - | - |
| Tx adherence | -2.91 | -7.54 | 1.73 | 0.22 | Tx adherence | -8.50 | -18.05 | 1.04 | 0.08 |
| % Male | -0.74 | -11.64 | 10.16 | 0.89 | % Male | **11.63** | **2.95** | **20.31** | **<0.01** |
| Mean age | 0.03 | -0.06 | 0.12 | 0.51 | Mean age | 0.03 | -0.02 | 0.09 | 0.27 |
| Baseline OSA | -0.01 | -0.06 | 0.03 | 0.53 | Baseline OSA | -0.04 | -0.09 | 0.01 | 0.13 |

**MIBG (n=2 comparisons):** Only 2 studies for this outcome, with no information on treatment adherence in either study. It was not possible to examine the impact of age and sex due to 1 study not reporting age and sex distribution in the sample. Meta-regression was not attempted.
